# Deficiency of muscle-generated brain-derived neurotrophic factor causes inflammatory myopathy through reactive oxygen species-mediated necroptosis and pyroptosis

**DOI:** 10.1016/j.redox.2024.103418

**Published:** 2024-11-08

**Authors:** Brian Pak Shing Pang, Elsie Chit Yu Iu, Miaojia Hang, Wing Suen Chan, Margaret Chui Ling Tse, Connie Tsz Ying Yeung, Mingfu Wang, Parco Ming Fai Siu, Chi Wai Lee, Keqiang Ye, Ho So, Chi Bun Chan

**Affiliations:** a School of Biological Sciences, Faculty of Science, The University of Hong Kong, Hong Kong Special Administrative Region; b Shenzhen Key Laboratory of Food Nutrition and Health, Institute for Advanced Study, Shenzhen University, Shenzhen, 518060, Hong Kong Special Administrative Region; c Division of Kinesiology, School of Public Health, The University of Hong Kong, Pok Fu Lam, Hong Kong Special Administrative Region; d Department of Biology, Hong Kong Baptist University, Hong Kong Special Administrative Region; e Faculty of Life and Health Sciences, and Brain Cognition and Brain Disease Institute (BCBDI), Shenzhen Institute of Advanced Technology, Chinese Academy of Sciences, Shenzhen, Hong Kong Special Administrative Region; f Department of Medicine & Therapeutics, Faculty of Medicine, The Chinese University of Hong Kong, Hong Kong Special Administrative Region; g State Key Laboratory of Pharmaceutical Biotechnology, The University of Hong Kong, Hong Kong Special Administrative Region

**Keywords:** BDNF, Muscle, ROS, Necroptosis, Pyroptosis

## Abstract

Idiopathic inflammatory myopathy (commonly known as myositis) is a group of immune-related diseases characterized by muscle damage, weakness, and fatigue with unknown causes. Although overactivated innate immunity is a widely believed cause of myositis onset, the mechanism that provokes and maintains a high immune response in myositis patients is still unclear. This study aims to test if brain-derived neurotrophic factor (BDNF) deficiency *per se* is sufficient to cause myositis and determine its underlying mechanism. We found that ablating BDNF production in skeletal muscle is sufficient to trigger myositis development in mice. Muscle-specific *Bdnf* knockout (MBKO) mice displayed extensive myocyte necrosis, mononuclear cell infiltration, and myophagocytosis. In association with these damages, elevated production of pro-inflammatory cytokines such as interleukin (IL) 23, IL-1β, IL-18, and tumor necrosis factor α (TNFα) was found in the muscle of MBKO mice. Disruption of sarcolemma integrity was also detected in MBKO mice, which is a result of necroptosis executioner Mixed lineage kinase domain-like protein (MLKL) and pyroptosis executioner Gasdermin D (GSDMD) activation. Mechanistically, diminishing BDNF synthesis in myotubes enhances the accumulation of mitochondrial reactive oxygen species (mtROS), which sensitizes the cells towards TNFα-induced receptor-interacting protein kinase (RIPs) activation and promotes the formation of NLR family pyrin domain containing 3 (NLRP3)-containing inflammasome. BDNF deficiency-induced cell death could be alleviated by scavenging mtROS, suppressing the activity of GSDMD, or inhibiting receptor-interacting kinase 3 (RIP3). Similarly, supplementation of BDNF mimetics, suppression of RIP3 activity, increasing the intramyocellular antioxidant, or enhancing mitophagy ameliorated the myopathies of MBKO mice and improved their muscle strength. Together, our study demonstrates that insufficient BDNF production in mouse muscle causes the development of pathological features of myositis via enhancing oxidative stress, necroptosis, and pyroptosis in myofibers.

## 1 Introduction

Idiopathic inflammatory myopathy (IIM), collectively called myositis, is a group of heterogeneous diseases affecting skeletal muscles. Based on the results of histopathological, clinical, and serological examinations, IIM is currently classified into five types – dermatomyositis (DM), immune-mediated necrotizing myopathy (IMNM), overlap myositis (OM), sporadic inclusion-body myositis (sIBM), and polymyositis (PM) [1]. The main clinical features of IIM include proximal muscle weakness, myalgia, myofiber necrosis, endomysial immune cell infiltration, high circulating concentrations of muscle enzymes, and the presence of circulating auto-antibodies in some cases.

Although the pathogenesis of IIM is not clear, it is generally believed that overactivation of autoaggressive immune cells is the root cause of the diseases, as evidenced by the success of glucocorticoid-based immunosuppressive therapies in ameliorating most disease symptoms. However, a significant number of patients are irresponsive to this mainstay treatment, suggesting that non-immune mechanisms in muscle may contribute to the disease pathogenesis [2]. For instance, several studies suggest that defective autophagy or disruption in mitochondrial dynamics promotes myofiber degeneration and muscle weakness in patients of some IIM subtypes [[3], [4], [5]]. In healthy skeletal muscles, mitochondria undergo constant remodeling through mitochondrial biogenesis, mitofusion, mitofission, and mitophagy (a selective form of autophagy that specifically targets mitochondria) to maintain cellular homeostasis. These cellular processes constitute an intricate quality control system to retain sufficient functional mitochondria for ATP production and eliminate damaged mitochondria that might trigger programmed cell death [6]. The damaged mitochondria are removed by two distinct but interconnected signaling pathways of mitophagy, which are driven by PTEN-induced kinase 1 (PINK1) kinase and Parkin ubiquitinase or by “mitophagy receptors” [7]. When mitophagy is disrupted, the accumulation of structurally/functionally damaged mitochondria might cause various adverse consequences such as ATP shortage, excessive production of reactive oxygen species (ROS), impaired fatty acid oxidation, and leakage of mitochondrial DNA, which are the intracellular factors for activating inflammatory responses [8].

In addition to apoptosis, mitochondrial dysfunction promotes necroptosis, which is a programmed pathway that results in lytic cell death and subsequent activation of an immune response [9]. Necroptosis is usually initiated by the activation of death receptors such as tumor necrosis factor α (TNFα) receptor 1, which triggers the formation of necrosome, a protein complex that contains receptor-interacting serine-threonine kinase 1 (RIP1) and RIP3 in the cell [10]. RIP3 in the necrosome phosphorylates the pseudokinase mixed lineage kinase domain-like (MLKL) and promotes its conformational changes to form membrane-associated oligomers, leading to the permeabilization of the plasma membrane, cell swelling, leakage of cellular content, and finally cell death [11]. RIP1 autophosphorylation is provoked by the high cellular ROS content, which enables RIP1 to recruit RIP3 and form a functional necrosome [12]. Because RIPs are proteolytic substrates of caspase 8 [13], necroptosis would only be activated when the cellular activity of caspase 8 is compromised, making it a backup mechanism to eliminate apoptosis-defective cells. Recent studies reported that necroptosis is an important contributor to myofiber cell death [14,15] but the initiator of myofiber necroptosis has not been identified.

Brain-derived neurotrophic factor (BDNF) is a member of the neurotrophins is essential for synaptic plasticity, neuronal survival, development, and differentiation [16]. By binding to its cognate receptor tropomyosin receptor kinase B (TrkB), BDNF promotes TrkB autophosphorylation to activate several signaling cascades, including phosphoinositide 3 kinase (PI3K)/Akt, Ras/extracellular signal-regulated kinase (ERK), and phospholipase Cγ (PLCγ)/cAMP responsive element binding protein (CREB) pathways [17]. BDNF is also a metabolic hormone that inhibits food intake [18,19]. Because of its prominent activity in regulating neuronal functions, most research on BDNF/TrkB signaling has mainly focused on its role in the central nervous system, whereas little attention has been paid to its functional activities in peripheral tissues like skeletal muscle. Nevertheless, several fragmented studies have shown that BDNF promotes muscle regeneration and myofiber differentiation [20,21], enhances intramyocellular lipid catabolism [22,23], and controls systemic glucose metabolism via triggering insulin synthesis [24]. Using the non-peptidyl BDNF-mimetic 7,8-dihydroxyflaonve (7,8-DHF), we have shown that chronic activation of TrkB in mouse muscle promotes mitochondrial biogenesis, triggers uncoupling protein 1 (*Ucp1*) expression, and increases systemic energy expenditure [[25], [26], [27]]. Indeed, BDNF in mouse skeletal muscle is essential to maintain metabolic homeostasis via an autocrine or paracrine manner as muscle-specific BDNF knockout (MBKO) mice displayed reduced AMP-activated protein kinase (AMPK) activity, leading to impaired autophagy and a failure of glucose-to-fatty acid utilization shift in the skeletal muscle during fasting [23]. BDNF is also a regulator of mitochondrial fission and mitophagy by promoting PINK1 and Parkin attachment to the mitochondria of myofibers, which is an essential mechanism to protect against obesity-impaired mitochondria turnover [28]. Hence, insufficient BDNF production leads to the accumulation of enlarged mitochondria with impaired respiratory capacity [28,29], which disrupts energy homeostasis in the tissue.

Given that mitochondrial dysfunction has been observed in the skeletal muscles of myositis patients [30], and that BDNF is a novel regulator of mitochondria clearance [28], insufficient BDNF production in skeletal muscle might cause muscle damage and weakness as observed in myositis patients. Nevertheless, the role of BDNF in myositis pathogenesis has not been examined and it remains unknown if BDNF content is reduced in the muscle of myositis patients. Therefore, this study aims to test if BDNF deficiency *per se* is sufficient to cause myositis-like muscle damage and determine its underlying mechanism.

## 2 Materials and methods

### 2.1 Chemicals and reagents

Recombinant human TNFα was purchased from Thermofisher (PHC3016). Palmitic acid was purchased from International Laboratory USA (1211550). 7,8-DHF was purchased from Tokyo Chemical Industry Co., Ltd (D1916). GSK872 was purchased from Cayman (23300). Disulfiram was purchased from MedChem Express (HY–B0240). Urolithin A was purchased from Sigma-Aldrich (USA). Antibodies against ASC (67824), cleaved-caspase 1 D296 (89332), cleaved-caspase 3 D175 (9661), caspase 3 (9662), cleaved-caspase 8 D387 (8592), p-ERK1/2 T202/Y204 (9106), ERK1/2 (9102), p-IRF3 S396 (29047), IRF3 (4302), p-JNK T183/Y185 (4668), JNK (9252), p–NF–κB p65 S536 (3033), NF-κB p65 (8242), NLRP3 (15101), p-RIP1 S616 (53286), RIP1 (3493), p-RIP3 T231/S232 (91702), RIP3 (15828), p-MLKL S345 (37333), MLKL (37705), and PARP (9542) were purchased from Cell Signaling Technology. Anti-BDNF (ab108319) and anti-TNFα antibodies (ab34674) were purchased from Abcam. Anti-GSDMD antibody (A10164) was purchased from Abclonal. Anti-tubulin antibody (T6074) was purchased from Sigma-Aldrich. Adenovirus that overexpresses shBdnf was synthesized by Cyagen (USA) based on a previously reported sequence [31].

### 2.2 Animal experiments

Muscle-specific *Bdnf* knockout (MBKO) mice were generated by crossing *Bdnf Flox/Flox* mice (Jackson Laboratory, 004339) with transgenic mice carrying the human *ACTA1*/α-skeletal actin-promoter-driven *Cre* (HSA-Cre; Jackson Laboratory, 006149). Genotyping was performed via PCR using genomic DNA extracted from the tail and using primers suggested by the Jackson Laboratory. Mice were housed in environmentally controlled conditions under a 12 h light/dark cycle with *ad libitum* access to standard rodent pellet food and water. All *in vivo* assays were approved by the Committee on the Use of Live Animals in Teaching and Research (CULATR) of the University of Hong Kong. Because of the sex-specific response in mitophagy [28], all *in vivo* experiments were performed on 1 to 18-month-old female mice.

Exercise training of the animals was performed using a five-lane treadmill with a variable speed motor for 4 weeks as described previously [23]. The muscle strength of the animals was tested using the grip strength meter (Bioseb, USA) as previously reported [23]. Briefly, mice were held by the tail, allowed to grasp the metal mesh of the meter with their four limbs, and then were pulled backward in the horizontal plane. The force applied to the mesh when the grasp was released was recorded as the peak tension. The test was repeated five consecutive times within the same session, and the mean value of all trials was presented as the grip strength of the animal.

*In vivo* Evans Blue staining was performed by injecting 1 % Evans Blue dye into the mice intraperitoneally. After 24 h, the mice were euthanized by anesthetic overdose, and the muscle tissues were collected, paraffin-embedded, sectioned, and imaged using an inverted fluorescence microscope.

### 2.3 7,8-DHF feeding and drug administration

The animals were randomly assigned to the control or the treatment groups. 7,8-DHF powder was dissolved directly in the drinking water to make a final concentration of 0.16 mg/ml. After 8 weeks of treatment, the mice were euthanized, and their tissues were collected.

GSK872 (0.75 mg/kg body weight/day) and Trolox (30/mg/kg kg body weight/day) were dissolved in sunflower oil and intraperitoneally injected into the mice. After 28 days of daily administration, the mice were euthanized, and their tissues were collected. Mice that have received i.p. injection of sunflower oil for 28 days were used as the control.

Prednisolone solution (5 mg/kg body weight/day) or saline was intraperitoneally injected into the mice. After 14 days of daily administration, the mice were euthanized, and their tissues were collected. H&E staining on the paraffin-embedded gastrocnemius sections was performed and used to quantify the number of mononuclear immune cell infiltration and myofiber necrosis.

Daily urolithin A (2.3 mg/kg/day) or sunflower oil was performed intraperitoneally in mice for 8 weeks as previously reported [32]. The mice were then euthanized, and their tissues and serum were collected for biochemical analysis.

### 2.4 Cell culture

C2C12 cells were purchased from the American Type Culture Collection (ATCC, CRL-1772) and cultured in DMEM (Life Technologies Ltd, 12,800,017) supplemented with 5 % FBS (Fisher Scientific, 10,309,433), 15 % calf serum (Fisher Scientific, 16–010159), 100 IU/mL penicillin, and 100 μg/mL streptomycin (Invitrogen, 15,140,122). Differentiation of myoblasts into myotubes was performed by incubating 100 % confluent myoblast with differentiating medium [2 % horse serum (Life Technologies Ltd, 16,050,130), 100 IU/mL penicillin, and 100 μg/mL streptomycin] for 4 days. RAW264.7 cells were purchased from ATCC (TIB-71) and cultured in RPMI 1640 medium (ThermoFisher Scientific, 11875093) supplemented with 10 % heat-inactivated FBS, 100 IU/mL penicillin, and 100 μg/mL streptomycin.

### 2.5 Macrophage migration assay

RAW264.7 cells were seeded on the transwell insert (Millipore, MCEP24H48) and co-cultured with C2C12 myotube for 24 h with or without anti-TNFα (5 μg/ml). On the next day, the inserts were removed, washed with PBS, and stained with 1 % crystal violet in methanol. Stained inserts were washed thoroughly with tap water. All the unmigrated RAW264.7 cells on top of the membrane inserts were wiped. The number of migrated macrophages on the bottom side was counted from three distinct regions per insert under a bright field microscope. Representative images from at least three independent assays are shown.

### 2.6 Cell staining and imaging

C2C12 myotubes were washed with Hanks’ Balanced Salt Solution (HBSS) before incubation of propidium iodide (10 μg/ml) or MitoROS 580 (Cayman, 25169) at 37 C for 30 min. The treated cells were then washed with HBSS twice and immediately proceeded to imaging. Cell imaging and fluorescence intensity detection were performed in BioTek Cytation 1 multimode reader (Agilent, United States).

### 2.7 Histological, immunohistochemical, and immunofluorescence staining

Gastrocnemius muscles were fixed in 4 % paraformaldehyde and paraffin embedding. Tissue sections were rehydrated in graded ethanol and stained with H&E using a commercially available kit (Abcam, USA).

Immunohistochemical staining was performed after antigen retrieval by citric acid. After blocking with BSA (ExCell Bio, BSA00500), the paraffin sections were stained with anti-CD4 (25229), anti-CD8 (98941), anti-CD206 (24595), anti-p62 (5114), anti-NLRP3 (15101) (Cell Signaling Technology), or anti-CD80 antibodies (Abclonal, A16039). Subsequent steps were performed using the Histostain®-SP Kits kit (Invitrogen, 95–9943) following manufacturer's instructions.

Immunofluorescence staining was performed by embedding tissues in Tissue-Tek O.C.T compound (Sakura Finetek USA Inc, OCT-4583) and frozen-sectioning. After fixation in 4 % paraformaldehyde, the tissues were blocked with BSA and stained with anti-p-RIP3 T231/S232 antibody (Cell Signaling Technology, 91702). Alexa Fluor 555-conjugated secondary antibodies (Thermo Fisher Scientific, A21426) was used to recognize the primary antibody. All images were visualized using LSM 780 confocal microscopy (Carl Zeiss, Germany). Representative results from at least three animals are shown.

### 2.8 Myofiber size quantification

The cross-sectional area (CSA) of muscle fibers was analyzed using H&E-stained sections. The CSA of each fiber was confined by the ‘Polygon selections’ tool of the computer program ImageJ, and the CSA was calculated automatically with the set scale. Three distinct regions from each section were imaged and counted to avoid selection bias, and 100 myofibers were measured in each region. Analysis was performed in a blinded manner.

### 2.9 Antibody array, enzyme activity assays, lipid peroxidation, and ELISA

Sera were collected for cytokine level detection using Proteome Profiler Mouse Cytokine Array Kit, Panel A (R&D Systems, ARY006) following manufacturer's instructions. The final blot development was exposed in the G:BOX Chemi XRQ Imager (Syngene, United Kingdom). Target spot intensity was measured by ImageJ with background subtracted. Creatine kinase activity in serum was measured with Creatine Kinase Activity Assay Kit (Abcam, ab155901). After stimulated with TNFα [33], H_2_O_2_ [34], or palmitic acid [35], lactate dehydrogenase activity in cell culture medium was measured using Cytotoxicity Detection Kit (Roche, 11644793001) following manufacturer's instructions. *In vitro* caspase-1 activity was measured by Caspase-Glo 1 Inflammasome Assay (Promega, G9951). Lipid peroxidation level of skeletal muscles was measured by Lipid Peroxidation (MDA) Assay Kit (Abbexa, abx096010) and normalized with total protein concentration. Muscular IL-1β (ab197742) and IL-18 (ab216165) were measured by ELISA (Abcam).

### 2.10 Real-time PCR

Total RNA was extracted by Trizol Isolation Reagent (Invitrogen, 10,296,028). First-strand cDNA was synthesized using Superscript III reverse transcriptase (Invitrogen, 11,904,018) and Oligo-dT17 as the primer. Real-time PCR was performed using SYBR green reagent (Bio-Rad Laboratories, 172,524) and detected by the LightCycler 96 (Roche Diagnostics, USA). The primer specificity has been verified by the NCBI BLAST to avoid the amplification of non-specific products. Information of the primers is shown in the Supplemental Table 1.

### 2.11 Immunoblotting

Tissue or cell extracts were prepared by homogenizing tissues in lysis buffer (50 mM Tris, pH 7.4, 40 mM NaCl, 1 mM EDTA, 0.5 % Triton X-100, 1.5 mM Na_3_VO_4_, 50 mM NaF, 10 mM Na_4_P_2_O_7_, 10 mM sodium β-glycerol phosphate, and protease inhibitor cocktail [Sigma-Aldrich, P8340]). Cell debris was removed by centrifugation, and the supernatants were collected for further analysis. Protein concentration of the lysates was determined by the Bio-Rad Protein Assay (Bio-Rad Laboratories, 5,000,006). An equal amount of protein lysate from each sample was loaded for SDS-PAGE. SDS-PAGE was performed using the mini-PROTEAN Tetra (Bio-Rad Laboratories, USA), and protein transfer was performed using the Trans-Blot Turbo Transfer System (Bio-Rad Laboratories, USA). Immunoblotting signals within the linear detection range were detected with a G:Box Chemi XRQ imager (Syngene, UK) and analyzed using ImageJ (NIH, USA). Representative results from three or more independent experiments are shown.

### 2.12 Statistical analysis

Results are expressed as the mean ± SEM and were considered significant when P < 0.05. Statistical tests performed were Student's t-test (independent variables: genotypes), one-way ANOVA (independent variables: inhibitors/drugs), or two-way ANOVA (independent variables: age and genotypes, drugs and genotypes, exercise and genotypes) followed by Tukey's multiple comparison test when the sample size between the test groups is equal or Bonferroni post-tests when the sample size between the test groups are unequal. Analyses were performed using GraphPad Prism (GraphPad Software, USA). The normal distribution of data and their equal variance in the *t*-test were measured by Kolmogorov-Smirnov Test and *F*-test, respectively (Supplemental Table 2).

## 3 Results

### 3.1 MBKO mice display pathological features of inflammatory myositis

**Fig. 1:**
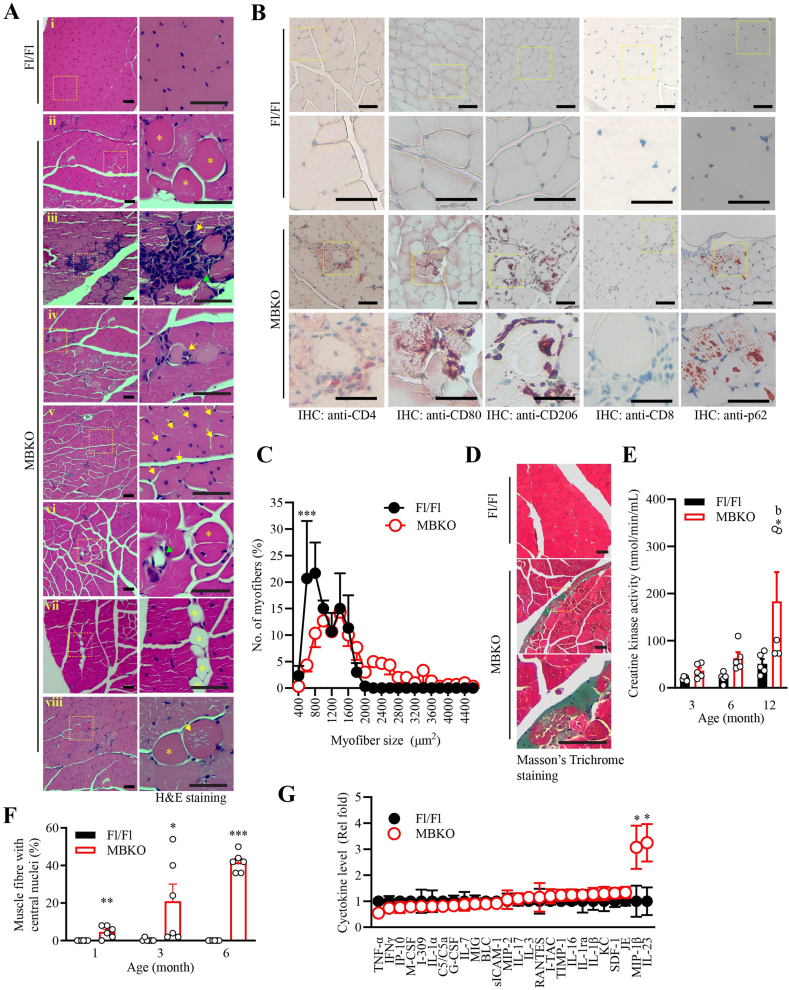
MBKO mice display pathological features of inflammatory myositis. A. Representative H&E staining images of gastrocnemius from female Fl/Fl and MBKO mice (6 months old). Myofiber oncosis (asterisks in ii and viii), mononuclear cell infiltration (arrow in iii), myophagocytosis (arrow in iv), presence of central nucleated myofiber (arrows in v), myofiber splitting (asterisk in vi), necrosis (green arrows in iii and vi), adipocyte replacement (asterisks in vii) and presence of intracellular vacuole (arrow in viii) were found exclusively in the muscle of MBKO mice. A magnified view of the dashed area is shown in the right panel. The scale bar represents 50 μm B. Immunohistological staining of gastrocnemius from female Fl/Fl and MBKO mice (6 months old). Haematoxylin was used for the nucleus counterstain. A magnified view of the dashed area is shown underneath the corresponding picture. The scale bar represents 50 μm C. Cross-sectional area of myofibers in the gastrocnemius of female Fl/Fl and MBKO mice (6 months old, ∗: P < 0.05, ∗∗∗: P < 0.001, two-way ANOVA, n = 6). D. Representative Masson's Trichrome staining images of gastrocnemius from female Fl/Fl and MBKO mice (6 months old). The magnified view of the dashed area is shown at the bottom panel. The scale bar represents 25 μm. E. Creatine kinase activity in the circulation of female Fl/Fl and MBKO mice (∗: P < 0.05 vs genotypes of the same age, b: P < 0.01 vs 3-month-old mice of the same genotype, two-way ANOVA, n = 5). F. The number of central-nucleated myofiber in the gastrocnemius of female Fl/Fl and MBKO mice (∗∗: P < 0.01, ∗∗∗: P < 0.001 vs genotypes of the same age, c: P < 0.001 vs 1-month-old mice of the same genotype, two-way ANOVA, n = 6). G. Cytokines content in the circulation of female BDNF Fl/Fl and MBKO mice (6-month-old) was detected by Cytokine Array (∗P < 0.05, Student's t-test, n = 6). (For interpretation of the references to colour in this figure legend, the reader is referred to the Web version of this article.)

We have shown previously that MBKO mice display muscle weakness, enhanced fatigability, and poor exercise endurance [23], suggesting a deficiency of local BDNF production might lead to muscle damage. Indeed, several histological markers of necrotizing muscle degeneration, including myofiber swelling (Fig. 1Aiii), mononuclear immune cell infiltration (Fig. 1Aiii), myofiber necrosis (Fig. 1Aiii), and myophagocytosis (Fig. 1Aiv) were observed exclusively in female MBKO mice. These pathological hallmarks could be detected in the female MBKO as early as 1 month old (Fig. S1A). In contrast, we could not detect these muscle damages, except for the presence of central nucleated myofibers, in the male MBKO mice (Fig. S1B). Hence, female MBKO mice were used in the subsequent analyses. Most endomysial infiltrated immune cells in MBKO muscle were lymphocytes and macrophages, as demonstrated by the positive staining for helper T cell marker (CD4) and activated macrophage markers (CD80 and CD206) (Fig. 1B). In contrast, CD8^+^ cytotoxic T cells were not detected, suggesting there was no autoimmune attack against the muscle fibers in MBKO mice (Fig. 1B). This notion was further supported by the comparable expression of antigens, including immunoreactive class I major histocompatibility complex (*H2kl*), high mobility group box 1 (*Hmgb1*), and T-cell co-stimulatory molecule CD28 (Fig. S1C), that can be detected in some 10.13039/501100007125IIM patients with high autoimmunity [36,37]. Because infiltrating macrophages and helper T cells are responsible for removing necrotic myofibers and promoting muscle recovery [38], extensive muscle regeneration was detected in the muscle of MBKO mice, which was demonstrated by the presence of numerous central nucleated myofibers (Fig. 1Av). These pathological changes are not a result of acute damage, as features of myopathic chronicity, including variation in muscle size (Fig. 1C), fiber splitting (Fig. 1Avi), fatty replacement (Fig. 1Avii), and mild fibrosis (Fig. 1D) were seen in the muscles of MBKO mice. Moreover, the creatine kinase activity (Fig. 1E) in the blood and the number of central nucleated myofiber (Fig. 1F) of MBKO mice increased with age, suggesting a progressive development of muscle damage and regeneration in the knockout mice. We also found intracellular vacuolation (Fig. 1Aviii) and autophagy receptor sequestosome-1 (p62) aggregation (Fig. 1B) exclusively in the muscle of MBKO mice, which is possibly a result of defective autophagy and mitophagy as we reported previously [28]. Moreover, higher circulating levels of myositis biomarkers, including interleukin 23 (IL-23) [39] and macrophage inflammatory protein [CC motif chemokine ligand 4 (CCL4)] [40], were detected in MBKO mice (Fig. 1G). These results indicate that a deficiency of muscle-generated BDNF induces the development of myositis. No skin ulcer nor abnormal histological structure, such as immune cell infiltration, was found in the skin of MBKO mice (Fig. S1D), suggesting BDNF deficiency in muscle did not induce the typical cutaneous manifestations of DM [41].

### 3.2 BDNF depletion in skeletal muscle promotes TNFα secretion to facilitate macrophage infiltration

**Fig. 2:**
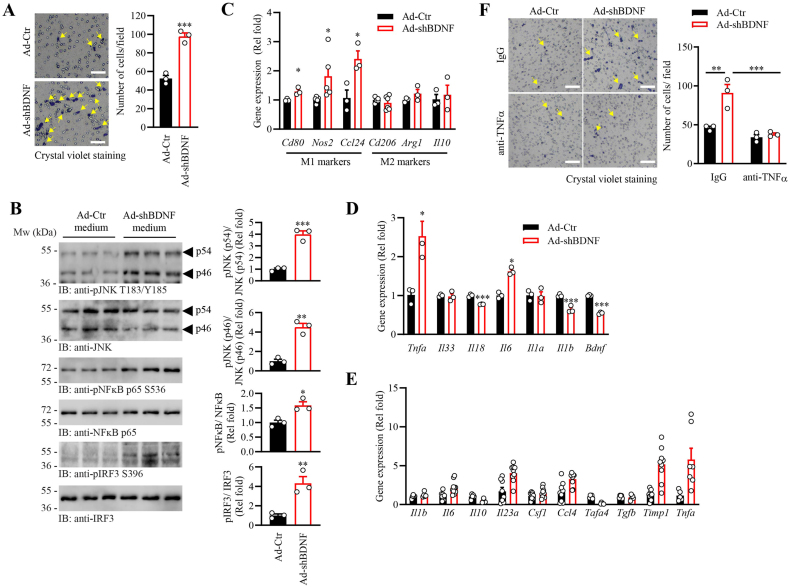
Insufficient BDNF synthesis in myofiber promotes macrophage activation. A. Transwell infiltration of cells in the macrophage-myotube co-culture. C2C12 myotubes were infected with Ad-Ctr or Ad-shBDNF and co-cultured with RAW264.7 macrophage for 24 h. The migrated macrophages across the transwell insert were stained and counted (∗∗∗: P < 0.01, Student's t-test, n = 3). Representative images of the transwell-insert membrane after crystal violet staining are shown on the left panel. The arrows indicate migrated macrophages and the scale bar represents 100 μm. B. Activation of the TNFα signaling in RAW264.7 cells after co-cultured with Ad-Ctr- or Ad-shBDNF-infected C2C12 myotubes for 24 h (∗: P < 0.05, ∗∗: P < 0.01, ∗∗∗: P < 0.001, Student's t-test, n = 3). C. Gene expression in the RAW264.7 cells after stimulated by culture medium collected from Ad-Ctr- or Ad-shBDNF-infected C2C12 myotubes for 24 h (∗: P < 0.05, Student's t-test, n = 3–6). D. Gene expression in the Ad-Ctr- or Ad-shBDNF-infected C2C12 myotubes (∗: P < 0.05, ∗∗∗: P < 0.001, Student's t-test, n = 3). E. Gene expression in the gastrocnemius of female Fl/Fl and MBKO mice (6 months old, ∗: P < 0.05, ∗∗: P < 0.01, ∗∗∗: P < 0.001, Student's t-test, n = 7–9). F. Neutralizing TNFα by anti-TNFα antibody abolished RAW264.7 migrations in the macrophage-myotube co-culture. C2C12 myotubes were infected with Ad-Ctr or Ad-shBDNF and co-cultured with RAW264.7 macrophage in the presence of IgG or anti-TNFα antibody for 24 h. The migrated macrophages across the transwell insert were stained and counted (∗∗: P < 0.01 vs IgG, c: P < 0.001 vs Ad-Ctr, two-way ANOVA, n = 3). Representative images of transwell insert membrane after crystal violet staining are shown on the left panel. The arrows indicate migrated macrophages and the scale bar represents 100 μm. (For interpretation of the references to colour in this figure legend, the reader is referred to the Web version of this article.)

To demonstrate that mononuclear immune cell infiltration in the skeletal muscle of MBKO mice is initiated by the myofiber, we suppressed *Bdnf* expression in C2C12 myotubes using adenovirus (Ad-shBDNF) infection and co-cultured with RAW264.7 macrophages in the transwell assay. When compared with the control-adenovirus (Ad-Ctr)-infected C2C12 myotubes, BDNF depletion in myotubes induced more macrophages migration across the transwell membrane (Fig. 2A). Augmented phosphorylation of the core inflammatory signaling molecules, including c-Jun N-terminal kinase (JNK) and nuclear factor kappa-light-chain-enhancer of activated B cells (NFκB), was detected in the macrophages co-cultured with Ad-shBDNF-infected C2C12 myotubes (Fig. 2B). Moreover, phosphorylation of interferon regulator factor 3 (IRF3), an activation marker of macrophage M1 polarization [42], was increased in the RAW264.7 cells after co-cultured with BDNF-knocked down myotubes (Fig. 2B), which was associated with a higher expression of M1 [CD80 (*Cd80*), nitric oxide synthase (*Nos2*) and *Ccl24*], but not M2 [CD206 (*Cd206*, arginase-I (Arg*1*) and *Il10*], polarization markers [43] (Fig. 2C). These results suggested that the activated inflammatory signaling and macrophage polarization were probably induced by the secretory factors from BDNF-deficient myotubes. Indeed, Ad-shBDNF-infection enhanced the expression of tumor necrosis factor α (TNFα), and its regulated gene IL-6 [33] in C2C12 myotubes (Fig. 2D) and the muscle of MBKO mice (Fig. 2E). In addition, a higher expression of *Il23* and *Ccl4* was detected in the muscle of MBKO mice, which aligned with the increased concentration of these cytokines in their blood (Fig. 1G). Interestingly, the circulating TNFα in MBKO mice was not elevated, suggesting the TNFα generated by the muscle of MBKO mice might only serve as an autocrine/paracrine to induce localized inflammatory response, which is supported by the elevation of its downstream target gene tissue inhibitor of metallopeptidase 1 (*Timp1*) in the muscle (Fig. 2E) [44]. Indeed, elevated TNFα content in the muscle biopsy of IIM patients does not correlate well with the TNFα concentration in their circulation [45,46], which is similar to our observation in the MBKO mice. To confirm that TNFα production from the BDNF-deficient myotubes is the dominant cause of macrophage activation in the co-culture system, we neutralized the function of TNFα by including anti-TNFα antibody in the culture medium. As anticipated, abolished RAW264.7 migration was detected when TNFα in the culture medium of Ad-shBDNF-infected myotubes was countervailed by the antibody (Fig. 2F). Together, our data indicated that the depletion of BDNF synthesis in skeletal muscles caused TNFα overproduction in myofibers, which recruits the macrophage to the tissue.

### 3.3 BDNF-deficient myotubes are hypersensitive to TNFα-induced cell death

**Fig. 3:**
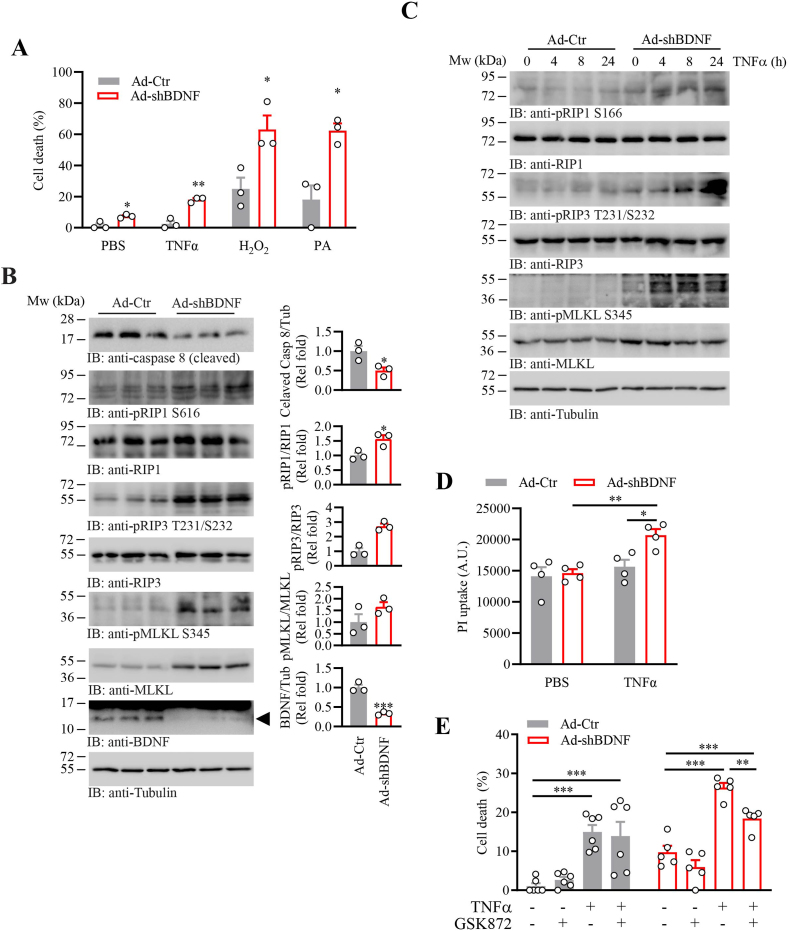
BDNF deficiency enhanced TNF-induced necroptosis in cultured myotubes. A. Cell death of Ad-Ctr or Ad-shBDNF-infected C2C12 myotube after stimulation by PBS, TNFα (100 ng/ml), H_2_O_2_ (1 mM), or palmitic acid (PA, 600 μM) for 24 h was assessed by lactate dehydrogenase (LDH) release assay (∗: P < 0.05, ∗∗: P < 0.01, Student's t-test, n = 3). B. Necroptosis signaling in the Ad-Ctr or Ad-shBDNF-infected C2C12 myotube was determined by immunoblotting. Quantification of the immunoblot is shown in the right panel (∗: P < 0.05, ∗∗: P < 0.01, ∗∗∗: P < 0.001, Student's t-test, n = 3). C. TNFα stimulation exaggerates the necroptosis signaling in BDNF-deficient C2C12 myotube. Ad-Ctr or Ad-shBDNF-infected C2C12 myotube after stimulation by TNFα (100 ng/ml) for various time intervals, and the necroptosis signaling was determined by immunoblotting. D. Uptake of propidium iodide (PI) in Ad-Ctr or Ad-shBDNF-infected C2C12 myotube with or without TNFα (100 ng/ml, 24 h) stimulation (∗: P < 0.05 vs Ad-Ctr, b: P < 0.01 vs PBS, two-way ANOVA, n = 4). E. Ad-Ctr or Ad-shBDNF-infected C2C12 myotubes were pretreated with RIP3 inhibitor GSK872 (1 μM) for 2 h, followed by TNFα (100 ng/ml) stimulation for 24 h. The amount of cell death was determined by LDH release assay (∗∗: P < 0.01, ∗∗∗: P < 0.001, one-way ANOVA n = 5–6).

Previous studies reported that TNFα promoted muscle cell death [47]. Consistent with the higher TNFα expression level, Ad-shBDNF-infected myotubes showed a higher spontaneous cell death than the control cells, which was further exaggerated in the presence of exogenous TNFα (Fig. 3A). The *Bdnf*-knocked down myotubes were also more fragile against oxidative stress (H_2_O_2_)- and lipotoxicity [palmitic acid (PA)]-induced cell demise (Fig. 3A). We did not observe increased cleavage of caspase 3 or poly(ADP-ribose) polymerase (PARP) in Ad-shBDNF-infected myotubes (Fig. S2), suggesting apoptosis signaling is not involved in cell death. In contrast, a lower amount of caspase 8 cleavage was detected in the *Bdnf* knocked-down myotubes than the control cells (Fig. 3B, 1st panel). Because caspase 8 inhibition is a pre-requisite of TNFα-mediated necroptosis [48], we examined the necroptosis signaling in myotubes after BDNF depletion. As anticipated, phosphorylation of the necroptosis mediator RIP1 and its downstream target RIP3 was higher in Ad-shBDNF-infected myotubes than in the control cells, which provided the necessary docking site to phosphorylate and activate MLKL [11]. Hence, the RIP3-mediated MLKL S345 phosphorylation was significantly increased in the BDNF-depleted myotubes (Fig. 3B). In addition, prolonged TNFα stimulation only provoked RIP1, RIP3, and MLKL phosphorylation in the Ad-shBDNF-infected myotubes but not the control cells (Fig. 3C), suggesting the absence of BDNF in myofiber sensitized the cells towards necroptosis when they were stimulated by TNFα. Concur with the fact that RIP3-mediated MLKL phosphorylation promotes its plasma membrane anchorage to compromise the membrane integrity [49,50], myotubes with *Bdnf* depletion displayed a higher influx of the non-permeable fluorescent dye propidium iodide (PI) into the myotubes after TNFα challenge (Fig. 3D). Moreover, the RIP-MLKL pathway is important to the deathly function of TNFα in BDNF-deficient myotubes as the presence of RIP3-specific inhibitor GSK872 [51] reduced the TNFα-induced cell death in the Ad-shBDNF-infected myotubes (Fig. 3E). In contrast, the TNFα-initiated cell demise was not affected by GSK872 in the Ad-Ctr-infected cells, suggesting BDNF-deficiency is the molecular switch that shifts the induction of cell death by TNFα towards the RIP-dependent necroptosis. Together, our data indicate that BDNF deficiency in myotubes promotes TNFα-induced necroptosis.

### 3.4 Augmented necroptosis and defective sarcolemma in the skeletal muscle of MBKO mice

**Fig. 4:**
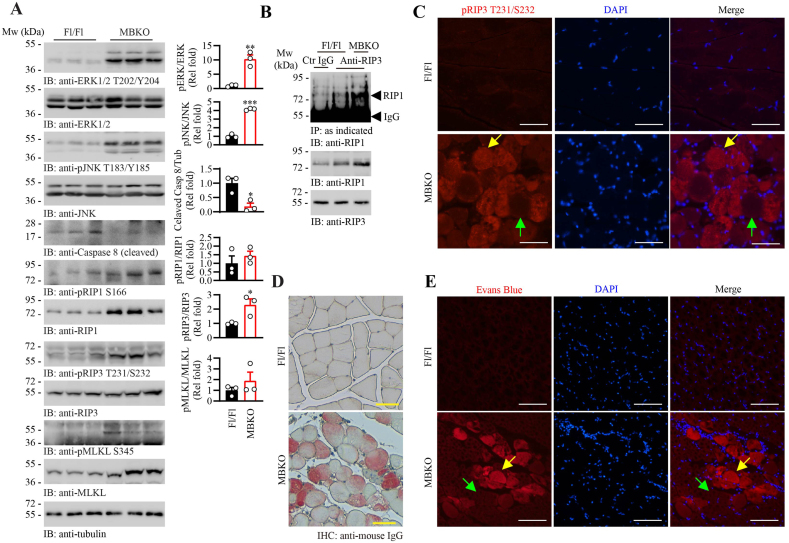
Enhanced necroptosis in the muscle of MBKO mice. A. Necroptosis signaling in the gastrocnemius of female Fl/Fl and MBKO mice (6 months old). Quantification of the immunoblot is shown in the right panel (∗: P < 0.05, ∗∗: P < 0.01, ∗∗∗: P < 0.001, Student's t-test, n = 3). B. The presence of necrosome in the gastrocnemius of female Fl/Fl and MBKO mice (6 months old) was determined by co-immunoprecipitation (top panel). The amount of RIP1 (middle panel) and RIP3 (bottom panel) input was also examined. C. Intramyocellular content of phosphorylated RIP3 in the gastrocnemius of female Fl/Fl and MBKO mice (6 months old) was examined by immunofluorescence staining. The yellow and green arrows indicate representative myofibers with high and low cellular content of phosphorylated RIP3, respectively. The scale bar represents 50 μm. D. IgG uptake in the gastrocnemius of female Fl/Fl and MBKO mice (6 months old) was determined by immunohistochemical staining. The scale bar represents 50 μm. E. Evans blue uptake in the gastrocnemius of female Fl/Fl and MBKO mice (6 months old) was determined by fluorescence microscopy. The yellow and green arrows indicate representative myofibers with ruptured and intact sarcolemma, respectively. The scale bar represents 50 μm. (For interpretation of the references to colour in this figure legend, the reader is referred to the Web version of this article.)

Consistent with the elevated pro-inflammatory cytokine expression in the gastrocnemius (Fig. 2E), the downstream signaling of cytokine activation, including ERK T202/Y204 and JNK T183/Y185 phosphorylation, was robustly increased in the MBKO mice (Fig. 4A). Similar to Ad-shBDNF-infected myotubes, we could not detect higher caspase 3 activation in the muscle of MBKO mice, although their muscles have more total caspase 3 protein than the Fl/Fl control (Fig. S2B). In contrast, a reduction of activated caspase 8 was found in the muscle of MBKO (Fig. 4A), which might explain the RIP1 accumulation in their tissue (Fig. 4A) because RIP1 is the proteolytic substrate of caspase 8 [52]. Furthermore, the amount of phosphorylated RIP1 (S166), RIP3 (T231/S232), and MLKL (345) was higher in MBKO muscle than that in Fl/Fl mice (Fig. 4A). This elevated RIP3 and MLKL phosphorylation could be observed in 1-month-old MBKO muscle (Fig. S3A). In contrast, no significant alternation in RIP3 and MLKL phosphorylation was detected in the adult male MBKO mice (Fig. S3B). The skeletal muscle of MBKO mice also contained a higher amount of necrosome than that of Fl/Fl control, as revealed by more RIP1-RIP3 complex in the MBKO muscle (Fig. 4B). Necroptosis mainly occurred in the myofiber of MBKO mice as immunoreactivity of T231/S322-phosphorylated RIP3 could only be detected in their myofibers (Fig. 4C). MBKO muscle also has a high serum IgG accumulation in their myofiber (Fig. 4D), which is a marker for muscle tissue necrosis [53], whereas no IgG was detected in the muscle of Fl/Fl control (Fig. 4D). Similarly, the accumulation of Evans blue, a widely used membrane impermeable dye for muscle damage assessment [54], was exclusively detected in the myofibers of MBKO mice, further implying the presence of necrosis in their muscle (Fig. 4E).

### 3.5 Deficiency of BDNF in muscle provokes ROS accumulation and pyroptosis

**Fig. 5:**
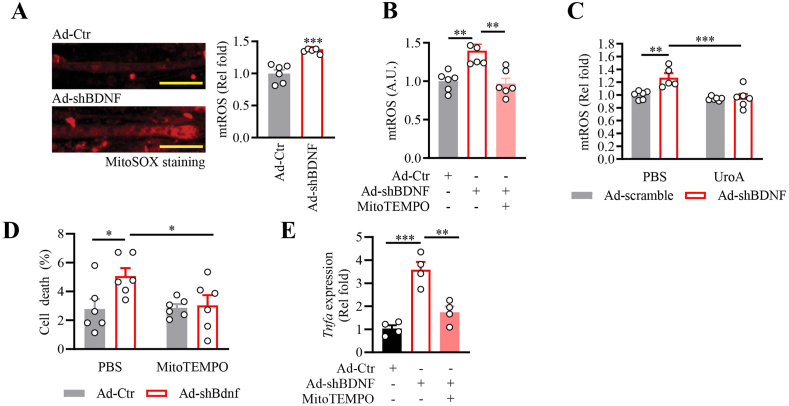
BDNF deficiency promotes mitochondrial ROS production. A. Mitochondrial superoxide in the Ad-Ctr or Ad-shBDNF-infected C2C12 myotubes was determined by MitoSox Red staining (∗∗∗: P < 0.001, Student's t-test, n = 6). The scale bar represents 100 μm. B. Mitochondrial superoxide in the Ad-Ctr or Ad-shBDNF-infected C2C12 myotubes treated with mitoTEMPO (15 mM, 24 h) was determined by MitoSox Red staining (∗∗: P < 0. 01, one-way ANOVA, n = 5–6). C. Mitochondrial superoxide in the Ad-Ctr or Ad-shBDNF-infected C2C12 myotubes treated with Urolithin A (50 μM, 24 h) was determined by MitoSox Red staining (∗∗: P < 0. 01, two-way ANOVA, n = 5–6). D. Ad-Ctr or Ad-shBDNF-infected C2C12 myotubes were treated with PBS or mitoTEMPO (15 mM, 24 h), and cell death was determined by the LDH release assay (∗: P < 0.01, ∗∗: P < 0.01, two-way ANOVA, n = 6). E. Expression of *Tnfa* in the Ad-Ctr or Ad-shBDNF-infected C2C12 myotubes treated with mitoTEMPO (15 mM, 24 h) was determined by real-time PCR (∗∗: P < 0.01, ∗∗∗: P < 0.001, One-way ANOVA, n = 4). (For interpretation of the references to colour in this figure legend, the reader is referred to the Web version of this article.)

Given that BDNF maintains mitochondrial dynamics in myotubes [28], depleting *Bdnf* expression in C2C12 myotubes causes elevated mitochondrial ROS content (Fig. 5A). The elevated ROS in BDNF-depleted myotubes decreased when the cells were treated with MitoTEMPO, a mitochondrial-targeted antioxidant (Fig. 5B), or enhanced mitophagy by urolithin A treatment [55] (Fig. 5C), further confirming the mitochondrial origin of the high cellular ROS. Moreover, myotube cell death (Fig. 5D) and *Tnfa* expression (Fig. 5E) caused by BDNF deficiency were abolished after MitoTEMPO treatment, demonstrating the predominant role of mtROS in causing various cellular abnormalities in BDNF-deficient muscle.

**Fig. 6:**
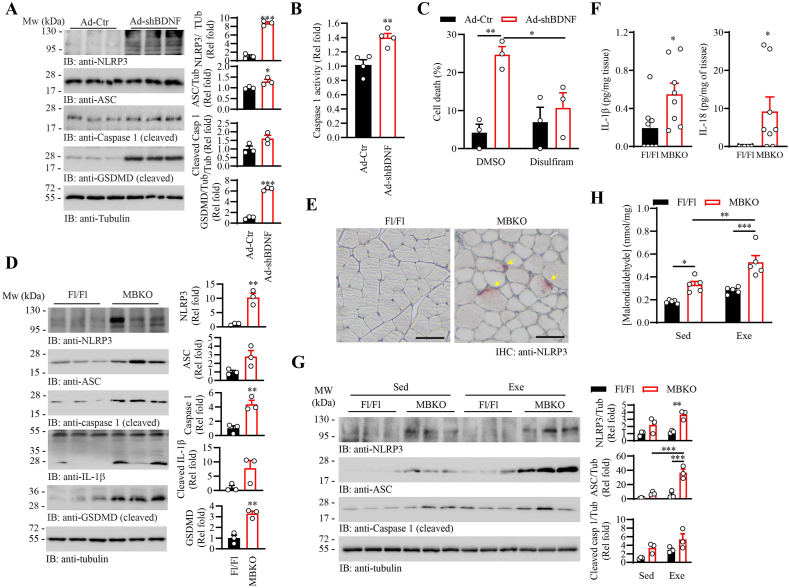
Enhanced pyroptosis in BDNF-deficient myotubes and the muscle of MBKO mice. A. Pyroptosis signaling in the Ad-Ctr or Ad-shBDNF-infected C2C12 myotubes was determined by Western blot analysis. Quantification of the immunoblot is shown in the right panel (∗: P < 0.05, ∗∗∗: P < 0.001, Student's t-test, n = 3). B. Enzymatic activity of caspase 1 in the Ad-Ctr or Ad-shBDNF-infected C2C12 myotubes (∗∗: P < 0.01, Student's t-test, n = 4). C. Ad-Ctr or Ad-shBDNF-infected C2C12 myotubes were treated with DMSO or disulfiram (1 μM), the inhibitor of pore formation by GSDMD, for 24 h. Cell death was determined by the LDH release assay (∗: P < 0.01, ∗∗: P < 0.01, two-way ANOVA, n = 3). D. Pyroptosis signaling in the gastrocnemius of female Fl/Fl and MBKO mice (6 months old) was determined by Western blot analysis. Quantification of the immunoblot is shown in the right panel (∗∗: P < 0.01, Student's t-test, n = 3). E. Immunohistochemical staining of NLRP3 in the gastrocnemius of female Fl/Fl and MBKO mice (6-month-old). Representative myofibers with accumulated NLRP3 are indicated by the arrows. The scale bar represents 50 μm. F. Intramyocellular content of IL-1β and IL-18 in gastrocnemius of female Fl/Fl and MBKO mice (6 months old) as determined by ELISA (∗: P < 0.05, Student's t-test, n = 8). G. Pyroptosis signaling in the gastrocnemius of sedentary (Sed) Fl/Fl and MBKO mice (7-month-old female) or those animals received chronic running training (Exe) as determined by Western blot analysis. Quantification of the immunoblot is shown at the bottom panel (∗∗: P < 0.01, ∗∗∗: P < 0.001, two-way ANOVA, n = 3). H. Intramyocellular malondialdehyde content in the gastrocnemius of female Fl/Fl and MBKO mice (7 months old) (∗: P < 0.05, ∗∗: P < 0.01, ∗∗∗: P < 0.001, two-way ANOVA, n = 5).

Studies have shown that high cellular ROS content promotes tissue damage by forming the supramolecular complex inflammasome, leading to programed pyroptotic cell death [56]. When compared with the Ad-Ctr-infected cells, depletion of BDNF in myotubes resulted in an accumulation of key inflammasome components, including nucleotide-binding oligomerization domain-like receptor 3 (NLRP3) and apoptosis-associated speck-like protein containing a CARD (ASC) (Fig. 6A). Activation of the inflammasome in the BDNF-deficient myotubes was further demonstrated by elevated cleavage of pro-caspase 1 into the mature form (Fig. 6A) and increased activity of caspase 1 (Fig. 6B), which aligned with the augmented cleavage of its substrate gasdermin D (GSDMD), the plasma membrane pore-forming executioner of pyroptosis [57] (Fig. 6A). When the formation of GSDMD pore was blocked by disulfiram [58], the LDH leakage in Ad-shBDNF-infected myotubes was significantly reduced (Fig. 6C), implying the pyroptotic GSDMD activation contributes to the spontaneous cell death in BDNF-deficient myotubes.

Augmented pyroptosis was also detected in the gastrocnemius of MBKO mice, as revealed by the higher amount of NLRP3, ASC, activated caspase 1, and cleaved GSDMD than that of the Fl/Fl control (Fig. 6D). Similar to the necroptotic pathway, elevated pyroptosis in MBKO mice is a young-onset event as augmented GSDMD cleavage could be detected in the gastrocnemius of 1-month-old MBKO mice (Fig. S3A). The formation of inflammasome mainly occurred in the myofiber of MBKO mice as an accumulation of NLRP3 was detected near the sarcolemma (Fig. 6E). In alignment with the elevated caspase 1 in the inflammasome complex, which is the major protease for cleaving of pro-IL-1β to form mature peptide [59], more IL-1β cleavage was detected in the muscle of MBKO mice (Fig. 6D), which concurred with the accumulation of IL-1β in their muscle (Fig. 6F). The intramyocellular concentration of mature IL-18, another substrate of caspase 1 [60], was also higher in MBKO mice than that of Fl/Fl control (Fig. 6F).

The accumulation of intramyocellular NLRP3 and ASC was further increased in the MBKO mice after chronic exercise (Fig. 6G). This is possibly a result of exacerbated oxidative stress in MBKO mice as higher content of lipid peroxidation end product, malondialdehyde, was detected in their muscle (Fig. 6H). In association with the exaggerated pyroptosis, severer muscle damage was detected in the MBKO mice (Fig. S4A). On the other hand, chronic exercise did not further increase the RIP3 phosphorylation in the muscle of MBKO mice (Fig. S4B). Unexpectedly, chronic exercise enhanced caspase 1 activation and MLKL phosphorylation in the Fl/Fl mice muscle, which was independent of NLRP3-inflammasome and RIP3 activation (Fig. 6G and S4B). Together, our data suggests that BDNF deficiency increases the ROS content in skeletal muscle, which promotes pyroptosis and interleukin production.

Muscle damage in MBKO mice was rectified by 7,8-dihydroxyflavone (7,8-DHF) supplementation, necroptosis inhibition, enhanced cellular antioxidant content, or elevated mitophagy.

**Fig. 7:**
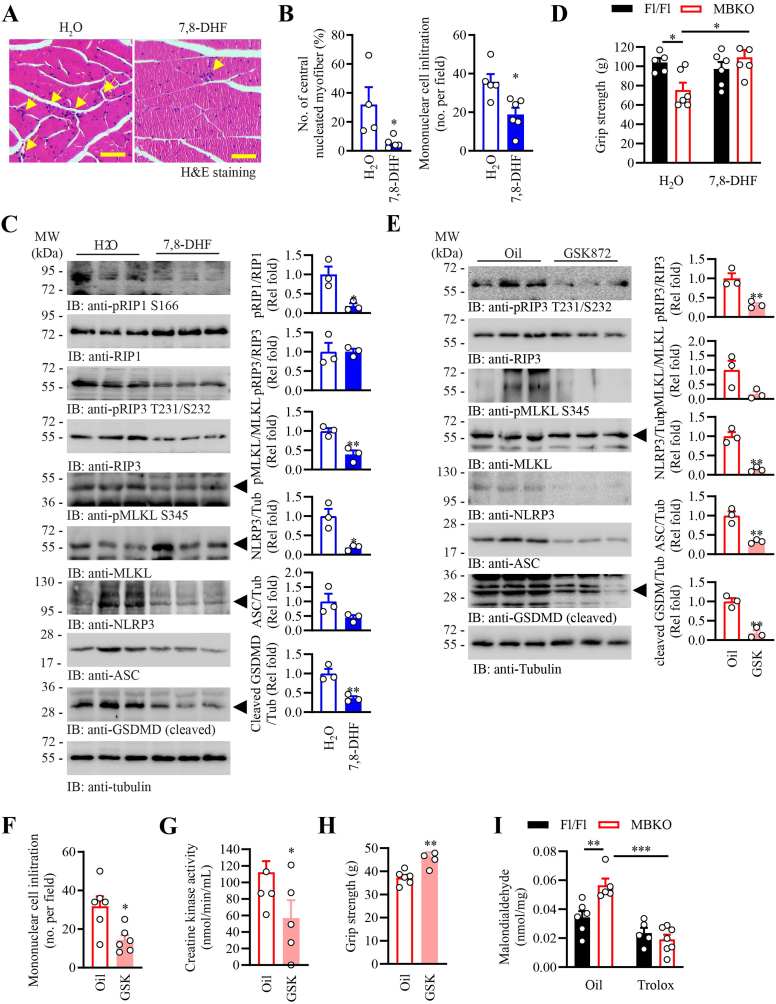

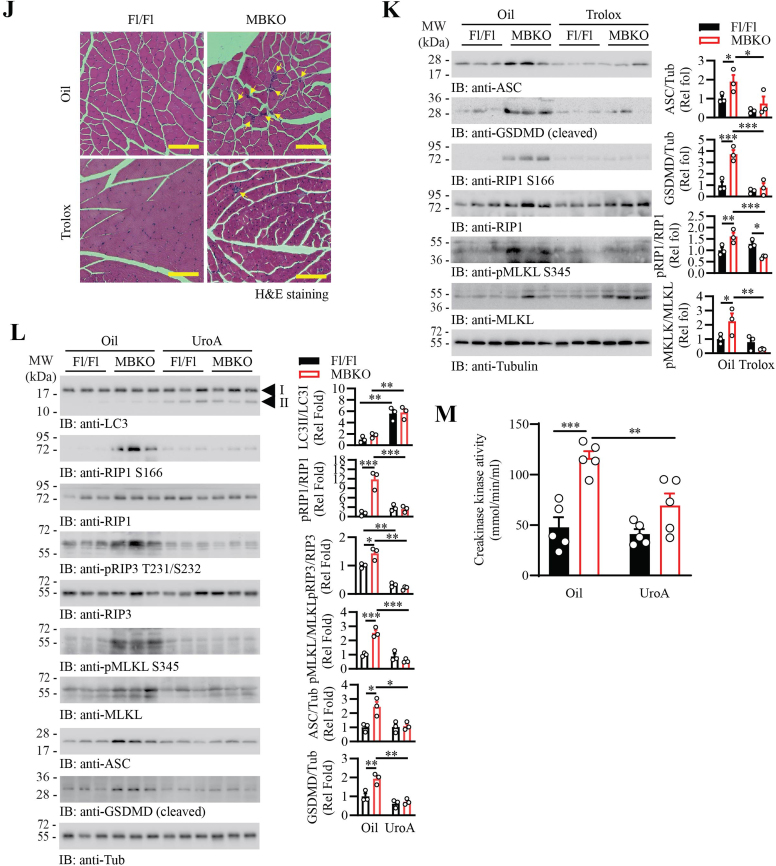
Reduction of muscle inflammation in MBKO mice by 7,8-DHF supplementation, necroptosis inhibition, antioxidant treatment, or mitophagy induction A. Representative H&E images of the gastrocnemius of female MBKO mice (9 months old) after H_2_O or 7,8-DHF treatment 3 months. The arrows indicate mononuclear cells infiltration. The scale bar represents 50 μm. B. The number of central-nucleated myofibers and mononuclear cell infiltration in the gastrocnemius of female MBKO mice (9 months old) after H_2_O or 7,8-DHF treatment 3 months (∗: P < 0.05, Student's t-test, n = 4–6). C. Necroptosis signaling in the gastro of female MBKO mice (9 months old) after H_2_O or 7,8-DHF treatment for 3 months was determined by Western blotting. Quantification of the immunoblot is shown in the right panel (∗: P < 0.05, ∗∗: P < 0.01, Student's t-test, n = 3). D. Grip strength of female Fl/Fl and MBKO mice (9 months old) after H_2_O or 7,8-DHF treatment (∗: P < 0.05, two-way ANOVA, n = 5–6). E. Necroptosis signaling in the gastro of female MBKO mice (7 months old) after daily administration of sunflower oil or RIP3 inhibitor GSK872 for 4 weeks. Quantification of the immunoblot is shown in the right panel (∗: P < 0.05, ∗∗: P < 0.01, Student's t-test, n = 3). F. The number of mononuclear cell infiltration in the gastrocnemius of female MBKO mice (7 months old) after daily administration of sunflower oil or RIP3 inhibitor GSK872 for 4 weeks (∗: P < 0.05, Student's t-test, n = 6) G. Creatine kinase activity in the serum of female MBKO mice (7 months old) after GSK872 treatment for 4 weeks (∗: P < 0.05, Student's t-test, n = 4–5). H. Muscle strength of female MBKO mice (7-month-old) after GSK872 treatment for 4 weeks (∗∗: P < 0.01, Student's t-test, n = 4–6). I. Intramyocellular malondialdehyde content in the gastrocnemius of female Fl/Fl and MBKO mice (7 months old) after sunflower oil or Trolox treatment for 4 weeks (∗∗: P < 0.01, ∗∗∗: P < 0.001, two-way ANOVA, n = 5–7). J. Representative H&E images of the gastrocnemius of female Fl/Fl and MBKO mice (9 months old) after sunflower oil or Trolox treatment for 4 weeks. The arrows indicate mononuclear cells infiltration. The scale bar represents 100 μm. K. Necroptosis and pyroptotic signaling in the gastro of female MBKO mice (7 months old) after sunflower oil or Trolox treatment for 4 weeks. Quantification of the immunoblot is shown in the right panel (∗: P < 0.05, ∗∗: P < 0.01, ∗∗∗: P < 0.001, two way-ANOVA, n = 3). L. Necroptosis and pyroptotic signaling in the gastro of female MBKO mice (4 months old) after sunflower oil or UroA treatment for 8 weeks. Quantification of the immunoblot is shown in the right panel (∗: P < 0.05, ∗∗: P < 0.01, ∗∗∗: P < 0.001, two way-ANOVA, n = 3). M. Creatine kinase activity in the serum of female MBKO mice (4 months old) after UroA treatment for 8 weeks (∗∗: P < 0.01, ∗∗∗: P < 0.001, two way-ANOVA, n = 5).

To verify if the myofiber necrosis in the MBKO mice was initiated by the over-activated immune response, prednisolone (PRDL, the first-line immunosuppressive drug used in treating IIM [61]), was applied to MBKO mice. Unexpectedly, PRDL treatment did not alleviate but worsened the muscle damage in MBKO mice, as revealed by a higher amount of mononuclear immune cell infiltration and myofiber necrosis in their skeletal muscle (Fig. S5A). The exacerbated myositis in MBKO mice after PRDL treatment was further demonstrated by a higher creatine kinase activity in their blood when compared with the control group (Fig. S5B). The necroptotic signaling, including RIP3 T231/S232 and MLKL S345 phosphorylation, was also higher in the muscle of PRDL-treated MBKO mice (Fig. S5C). These outcomes were not a result of PRDL ineffectiveness because the expression of the PRDL-responsive gene, fibroblast growth factor 21 (*Fgf21*) [62], was significantly increased in the liver of PDL-treated MBKO mice (Fig. S5D). Presumably, the inflammatory features observed in the MBKO muscle were initiated by the intrinsic defects of their myofibers, and the infiltration of immune cells provides a resolution to clear the damaged cell. To test this hypothesis, we restored the BDNF signaling in MBKO mice by feeding the animals with the bioavailable BDNF mimetics 7,8-DHF for 3 months [26]. As anticipated, a lower number of central nucleated myofiber and mononuclear immune cell infiltration was observed in the 7,8-DHF-treated MBKO mice (Fig. 7A and B). While 7,8-DHF treatment did not alter the pyroptosis and necroptosis signaling in the muscle of Fl/Fl mice (Fig. S6), reduced phosphorylation of RIP1 S166, RIP3 T231/S232, MLKL S345, and diminished accumulation of NLRP3, ASC, and cleaved GSDMD was detected in the muscle of MBKO mice after 7,8-DHF treatment, implying the pathological necroptosis and pyroptosis were corrected (Fig. 7C). In association with reduced necroptotic and pyroptotic signaling, the muscle strength of MBKO mice was improved after 7,8-DHF treatment (Fig. 7D).

To demonstrate that dysregulated necroptosis is a driver of myositis in MBKO mice, we pharmacologically inhibited RIP3 in the knockout animals by GSK872 administration. A significant reduction of necroptosis signaling, including RIP3 T231/S232 and MLKL S345 phosphorylation, was detected in the MBKO mice after GSK872-treatment (Fig. 7E), which was associated with a reduction of immune cells infiltration in their muscle (Fig. 7F). Interestingly, GSK872 treatment also diminished the pyroptosis signaling in the muscle of MBKO mice, as revealed by the lowered intramyocellular content of NLRP3 and ASC (Fig. 7E). The improvement of muscle damage in GSK872-treated MBKO mice was further revealed by the reduction of creatine kinase activity in their circulation (Fig. 7G) and augmented muscle strength of MBKO mice (Fig. 7H).

We also tested if scavenging the ROS in the muscle of MBKO mice could rescue the myositis phenotypes. After treating MBKO mice with Trolox, a vitamin E analog with potent antioxidant activity, the lipid oxidation in their muscle was significantly reduced (Fig. 7I). The muscle inflammation in MBKO mice was also improved, which was revealed by the lower number of immune cell infiltration (Fig. 7J). Moreover, the myocellular content of ASC, cleavage of GSDMD, and phosphorylation of RIP1 and MLKL were all significantly reduced in MBKO mice after Trolox treatment (Fig. 7K). These data confirm that excessive ROS is the driving cause of necroptosis and pyroptosis in MBKO mice.

Lastly, we verified if the suppressed mitophagy in the BDNF-deficient muscle is a major cause of augmented pyroptosis and necroptosis. Concur with the previous report [55], administration of the mitophagy enhancer, Urolithin A (UroA), for 8 weeks induced more LC3 lipidation in the muscle of Fl/Fl and MBKO mice (Fig. 7L, 1st panel). Moreover, the necroptosis pathway, including RIP1 S166, RIP3 T321/S232, and MLKL S345 phosphorylation, was significantly inhibited in the muscle of MBKO mice after UroA administration (Fig. 7L, 2nd to 9th panels). Similarly, UroA injection reduced the cellular content of ASC and GSDMD cleavage in the muscle of MBKO mice but not in that of the Fl/Fl control (Fig.s. 7L, 8th and 9th panels). Aligned with the reduced MLKL phosphorylation and GSDMD cleavage, the muscle damage in MBKO mice was mitigated, as revealed by the lower creatine kinase in their circulation (Fig. 7M). These data suggest that insufficient mitophagy is a major cause of the elevated necroptosis/pyroptosis pathway and muscle damage in the MBKO mice.

## 4 Discussion

Although the cause of IIM is poorly understood, it is generally believed that the attack of aggressive autoimmune cells contributes significantly to muscle damage. The unresponsive effect after immunosuppressive therapy in some patients, however, suggests that other dysregulated cellular activity in the myofiber might also cause the disease phenotypes. This notion is supported by the findings that endoplasmic reticulum (ER) stress, dysregulated sarcolemmal Ca^2+^ homeostasis, impaired autophagy, elevated ROS, augmented pyroptosis, and uncontrolled necroptosis were found in the muscle of some IIM patients [2,15,63]. Nevertheless, the cause of these endogenous cellular malfunctions remains unknown. In this study, we found that deficient BDNF production in myofibers resulted in excessive ROS accumulation and initiation of intricate cellular responses that lead to muscle damage and inflammation. Our data propose that blockage of BDNF synthesis promotes the production of TNFα (Fig. 2) and suppresses caspase 8 activation (Fig. 3, Fig. 4), hence sensitizing the myofibers towards TNFα-provoked necroptotic damage. Enhanced TNFα might also induce *Nlrp3* expression [64], which was subsequently activated by the elevated ROS in the muscle of MBKO mice to form an inflammasome platform, leading to the maturation of functional ILs and the pore-forming GSDMD for membrane perforation (Fig. 5). Eventually, the leakage of immunogenic content through the oligomerization of MLKL and GSDMD, and the production of inflammatory ILs might recruit the immune cells to induce inflammatory responses. In support of this proposed model, inhibition of necroptosis and pyroptosis in myofibers by disulfiram and GSK872 or increasing the cellular content of antioxidants reduces myocyte death and improves the muscle strength of MBKO mice (Fig. 7). Together, our results strongly suggest that BDNF deficiency in skeletal muscle is an unrecognized cause of necroptotic and pyroptotic cell damage, leading to the development of myositis.

Elevated TNFα has been found in IIM patients, but the cause and consequence of TNFα overproduction in their muscles have not been explored. Peng et al. recently reported that TNFα induced muscle cell death in the presence of a pan-caspase inhibitor, and the TNFα-induced necroptosis might be a cause of myofiber death in IIM [15]. In this study, we found that BDNF is a regulator of *Tnfa* expression and a molecular switch to initiate the TNFα-mediated necroptotic pathway. Interestingly, BDNF and TNFα are reciprocally regulated hormones in many tissues. While TNFα stimulates *Bdnf* expression in monocytes, astrocytes, neurons, and smooth muscle cells [[65], [66], [67], [68]], there are limited studies to elucidate the effect of BDNF on *Tnfa* expression. Nevertheless, some reports suggest that BDNF is a suppressor of *Tnfa* and counteracts the devasting impacts of TNFα *in vitro* [69,70]. A recent study demonstrated that the LPS-induced *Tnfa* expression was exaggerated in the heterozygous BDNF knockout mice [71], further suggesting BDNF is critical in regulating *Tnfa* expression. The detailed mechanism of how BDNF deficiency promotes *Tnfa* expression remains unknown at the current stage, but it is tempting to hypothesize that the high cellular ROS in *Bdnf* knocked down cells is a potential triggering factor [72], which is supported by our findings that mitoTEMPO treatment suppressed the *Tnfa* expression in the *Bdnf*-knocked down myotubes (Fig. 5). It is also noteworthy that TNFα is an inducer of mitochondrial ROS production and an inhibitor of PINK1-dependent mitophagy [73,74]. Hence, the elevated TNFα production in the muscle of MBKO mice might form a vicious cycle that perpetually enhances oxidative stress and muscle damage.

Numerous studies have proved that uncontrolled elevation of intramyocellular ROS has a negative impact on tissue metabolism and function. Hence, elevated ROS has been associated with a lot of myopathies, including IIM [75]. Meyer et al. further proposed that the elevated ROS content in the muscles of DM patients contributes to muscle damage and immune cell infiltration [76], but the underlying mechanism of ROS-induced muscle damage in myositis has not been explored. We have recently shown that muscle-derived BDNF is essential to maintaining mitochondrial dynamics via calcium/calmodulin-dependent protein kinase kinase (CamKK)-AMPK activation, and inhibiting BDNF synthesis in muscle leads to the accumulation of mitochondria with lower oxidative phosphorylation capacity [28]. Since impaired oxidative phosphorylation is a major cause of ROS accumulation in muscle, it is reasonable to find that insufficient BDNF synthesis *per se* is sufficient to cause ROS accumulation in the muscle cells (Fig. 5), which subsequently triggers pyroptosis as ROS elimination by antioxidant administration was able to reduce myofiber death (Fig. 6) and ameliorate the pyroptosis signaling (Fig. 7). Align with the findings that ROS promotes RIP1 autophosphorylation and the subsequent necrosome formation in fibroblast [12], RIP1 phosphorylation in the MBKO mice was reduced after Trolox treatment (Fig. 7), suggesting ROS is a universal inducer of necroptosis in different tissues. Presumably, supplementation of antioxidants in the muscles of IIM patients with overactivated signaling in pyroptosis, necroptosis, or both might be helpful in alleviating the inflammation.

Both necroptosis and pyroptosis lead to lytic cell death in a number of diseases, including ischemic cardiovascular damage, alcoholic liver disease, and Alzheimer's disease [77]. Recent studies have also suggested that augmented necroptosis and pyroptosis are the pathological features of IIM and Duchenne muscular dystrophy [14,15,53,63]. However, the triggering factor of necroptosis and pyroptosis in myopathy patients remains unknown. Our study demonstrates that BDNF deficiency is a common initiator of these damaging mechanisms in skeletal muscle via increasing ROS production. Our data also suggests that elevated necroptosis is linked to pyroptosis in muscle because pharmacological inhibition of RIP3 diminished the pyroptosis signaling in MBKO mice (Fig. 7). Indeed, necroptosis and pyroptosis are intertwined pathways that often crosstalk with each other in immune cells but its functional interplay in skeletal muscle has not been demonstrated. For instance, activation of the necroptotic MLKL complex increases the K^+^ efflux, which is an activator of inflammasome formation [78]. The crosstalk of necroptosis and pyroptosis in immune cells can also be induced by pathogen invasion, which forms a flexible cell death system with in-built fail-safe processes that protect the host from intracellular infections [79].

In addition to pharmacological treatment, chronic exercise has been proposed as an effective management to alleviate myositis symptoms and improve the muscle functions of IIM [80]. It has also been suggested that exercise protects against pyroptosis-mediated inflammation [81]. However, our data shows that chronic exercise exaggerated the pyroptosis phenotypes in MBKO mice. It is possible that the accumulation of functionally impaired mitochondria in the muscle of MBKO mice could not fully metabolize the nutrients during exercise, which generated an abnormally high amount of ROS, thus triggering more pyroptosis signaling in their muscle. Hence, genetic deposition or metabolic status like obesity [28] that might result in low intramyocellular BDNF content should be considered potential risk factors for muscle inflammation when the subject faces repetitive exercise. Because BDNF is an exercise-induced myokine [82], presumably, BDNF production in the muscle during or after exercise represents a protective mechanism to avoid excessive ROS-mediated pyroptotic cell death.

Protein aggregation associated with neurodegenerative diseases, such as β-amyloid (Aβ), β-amyloid precursor protein (βAPP), and α-synuclein, has been detected in the myofibers of patients with sIBM [83]. These proteins might disrupt muscle innervation during development because βAPP overexpression in myotubes prevents the formation of neuromuscular junction (NMJ) in the myotube-neuron coculture [84]. A recent study further shows that NMJ number is significantly reduced in the muscle of sIBM patients [85]. Immunohistological investigations have revealed a high accumulation of these proteins at the postsynaptic domain of the NMJ in sIBM patients [86,87], indicating that dysregulated protein accumulation may compromise mature NMJ function. Interestingly, several studies demonstrated that BDNF is important in NMJ development and transmission. It has been reported that BDNF production in muscle is crucial for NMJ formation by eliminating the overproduced neuromuscular contact in the polyinnervated muscle during development [88]. However, findings from the MBKO mice challenge this notion, as these mice exhibited normal NMJ structure and number, despite smaller motor endplate volume [21]. Nevertheless, the BDNF-TrkB signaling is essential for maintaining the NMJ function by facilitating the acetylcholine release through novel protein kinase C (nPKC) and myristoylated alanine rich C kinase substrate (MARCKS) [89]. Furthermore, TrkB is highly localized in the post-synaptic acetylcholine receptor (AChR)-rich motor-endplate, and eliminating TrkB in the motor endplate resulted in disassembly of post synaptic AChR clusters [90]. It is noteworthy that NMJ transmission in IIM patients or animal models has not been examined. Hence, further investigation on the potential role of BDNF signaling in NMJ function among myositis patients should be performed in the future, which could provide insights into the neurological consequences of BDNF deficiency in myositis.

In conclusion, our study demonstrates that inadequate BDNF production in mouse muscle contributes to the development of pathological features of myositis via enhancing oxidative stress, necroptosis, and pyroptosis in myofibers. Studies to determine the BDNF content in the skeletal muscle of myositis patients is warranted to further confirm the causative role of BDNF in myositis onset.

## CRediT authorship contribution statement

**Brian Pak Shing Pang:** Writing – review & editing, Writing – original draft, Methodology, Formal analysis. **Elsie Chit Yu Iu:** Methodology. **Miaojia Hang:** Methodology. **Wing Suen Chan:** Methodology. **Margaret Chui Ling Tse:** Methodology. **Connie Tsz Ying Yeung:** Methodology. **Mingfu Wang:** Formal analysis. **Parco Ming Fai Siu:** Formal analysis. **Chi Wai Lee:** Formal analysis. **Keqiang Ye:** Formal analysis. **Ho So:** Funding acquisition, Formal analysis, Conceptualization. **Chi Bun Chan:** Writing – review & editing, Writing – original draft, Visualization, Validation, Supervision, Resources, Project administration, Methodology, Investigation, Funding acquisition, Formal analysis, Data curation, Conceptualization.

## Declaration of competing interest

The authors declare that they have no known competing financial interests or personal relationships that could have appeared to influence the work reported in this paper.

## Data Availability

Data will be made available on request.
