# Supplementary material for: Deficiency of muscle-generated brain-derived neurotrophic factor causes inflammatory myopathy through reactive oxygen species-mediated necroptosis and pyroptosis
Source: Redox Biol. 2024 Nov 8;78:103418. doi: 10.1016/j.redox.2024.103418 (PMC11602578; doi:10.1016/j.redox.2024.103418)
Supplement: Multimedia component 1 [file mmc1.docx]

Table S1 Primer information

| **Gene name** | **GenBank accession number** | **Sequence** | | **Amplicon size (bp)** | **Tm (°C)** | |
| --- | --- | --- | --- | --- | --- | --- |
|  |  | **Forward (5' to 3')** | **Reverse (3' to 5')** |  | **Forward** | **Reverse** |
| *Arg1* | U51805 | AGAGATTATCGGAGCGCCTT | TTTTTCCAGCAGACCAGCTT | 94 | 57.1 | 56.6 |
| *Bdnf* | EF125673 | GCGGCAGATAAAAAGACTGC | GCAGCCTTCCTTGGTGTAAC | 141 | 56.1 | 58.2 |
| *Cat* | AY040626 | CCGACCAGGGCATCAAAA | GAGGCCATAATCCGGATCTTC | 74 | 58.2 | 60.2 |
| *Ccl4* | NM_013652 | CATGAAGCTCTGCGTGTCTG | GAAACAGCAGGAAGTGGGAG | 109 | 57.6 | 58.3 |
| *Ccl24* | NM_019577 | ATTCTGTGACCATCCCCTCAT | TGTATGTGCCTCTGAACCCAC | 187 | 60.3 | 61.6 |
| *Cd80* | BC145843 | TTCGTCTTTCACAAGTGTCTTCA | TGCCAGTAGATTCGGTCTTCA | 127 | 59.4 | 60.3 |
| *Cd206/Mrc1* | NM_008625 | CATTCCCTCAGCAAGCGATG | GGGTTCCATCACTCCACTCA | 272 | 58.2 | 59.3 |
| *Csf1* | NM_001113530 | CGACATGGCTGGGCTCCC | CGCATGGTCTCATCTATTAT | 256 | 63.9 | 50.3 |
| *Fgf21* | AB021975 | ACCTGGAGATCAGGGAGGAT | GCACAGGAACCTGGATGTCT | 131 | 59.3 | 59 |
| *Il1a* | NM_010554 | ACGTCAAGCAACGGGAAGAT | AAGGTGCTGATCTGGGTTGG | 124 | 58.7 | 59.5 |
| *Il1b* | M15131 | CCCAAGCAATACCCAAAGAAA | GCTTGTGCTCTGCTTGTGAG | 142 | 58.4 | 57.8 |
| *Il6* | J03783 | GGCCTTCCCTACTTCACAAGTCCG | TGCACAACTCTTTTCTCATTTCCACGA | 143 | 66.7 | 63.2 |
| *Il10* | M37897 | GGTTGCCAAGCCTTATCGGA | ACCTGCTCCACTGCCTTGCT | 191 | 59.7 | 62.5 |
| *Il18* | NM_001357222 | GCCATGTCAGAAGACTCTTGCGTC | GTACAGTGAAGTCGGCCAAAGTTGTC | 122 | 64.4 | 64.4 |
| *Il23a* | AF301619 | CCAGCAGCTCTCTCGGAATCT | ACTGCTGACTAGAACTCAGGC | 371 | 63.1 | 60.3 |
| *Il33* | NM_133775 | TCCAACTCCAAGATTTCCCCG | CATGCAGTAGACATGGCAGAA | 120 | 62.9 | 58.8 |
| *Nos2* | NM_010927 | CCGAAGCAAACATCACATTCA | GGTCTAAAGGCTCCGGGCT | 101 | 58.5 | 61.6 |
| *Rpl7* | BC051261 | CAAGAAGCGGATTGCCTTGA | TAACTTGAAGGGCCACAGGAA | 151 | 57.5 | 61.2 |
| *Sod1* | BC002066 | CAGGACCTCATTTTAATCCTCAC | TGCCCAGGTCTCCAACAT | 78 | 58.2 | 58.4 |
| *Sod2* | NM_013671 | GGCCAAGGGAGATGTTACAA | GAACCTTGGACTCCCACA | 216 | 57.1 | 57.4 |
| *Tafa4* | AY325123 | CCTATGTGTTAATGGTCTGCTGT | CCACCACTTCTCAATCACAATGG | 248 | 58.5 | 61.0 |
| *Tgfb* | M13177 | GACCGCAACAACGCCATCTA | GGCGTATCAGTGGGGGTCAG | 236 | 59.7 | 62.5 |
| *Timp1* | NM_011593 | GGCATCCTCTTGTTGCTATCACTG | GTCATCTTGATCTTATAACGCTGG | 170 | 61.9 | 57.6 |
| *Tnfa* | M13049 | CCTGTAGCCCACGTCGTAGC | TTGACCTCAGCGCTGAGTTG | 374 | 61.7 | 59.3 |
