# Supplementary material for: Deficiency of muscle-generated brain-derived neurotrophic factor causes inflammatory myopathy through reactive oxygen species-mediated necroptosis and pyroptosis
Source: Redox Biol. 2024 Nov 8;78:103418. doi: 10.1016/j.redox.2024.103418 (PMC11602578; doi:10.1016/j.redox.2024.103418)
Supplement: Multimedia component 2 [file mmc2.docx]

Table S2 Kolmogorov-Smirnov Test and F-test

|  |  | P value | |
| --- | --- | --- | --- |
| Figure | Measurement | Kolmogorov-Smirnov Test | *F*-Test |
| Fig 2A | Number of cells | 0.100 | 0.8697 |
| Fig 2B | pJNK p54 | 0.100 | 0.1786 |
|  | pJNK p46 | 0.100 | 0.4472 |
|  | pNFkB | 0.100 | 0.6132 |
|  | pIRF3 | 0.100 | 0.1002 |
| Fig 2C | *Cd80* | 0.100 | 0.2346 |
|  | *Nos2* | 0.100 | 0.0003 |
|  | *Cd24* | 0.100 | 0.9948 |
|  | *Cd206* | 0.100 | 0.0381 |
|  | *Arg1* | 0.100 | 0.1761 |
|  | *Il10* | 0.100 | 0.4088 |
| Fig 2D | *Tnfa* | 0.100 | 0.1733 |
|  | *Il33* | 0.100 | 0.1057 |
|  | *Il18* | 0.100 | 0.0383 |
|  | *Il16* | 0.100 | 0.8377 |
|  | *Il1a* | 0.100 | 0.6288 |
|  | *Il1b* | 0.100 | 0.3232 |
|  | *Bdnf* | 0.100 | 0.6457 |
| Fig 2E | *Il1b* | 0.100 | 0.4741 |
|  | *Il6* | 0.083 | 0.1160 |
|  | *Il10* | 0.100 | 0.9747 |
|  | *Il23a* | 0.100 | 0.5053 |
|  | *Csf1* | 0.100 | 0.2798 |
|  | *Ccl4* | 0.100 | 0.2916 |
|  | *Tafa4* | 0.100 | 0.1667 |
|  | *Tgfb* | 0.100 | 0.3447 |
|  | *Tmp1* | 0.100 | 0.0020 |
|  | *Tnfa* | 0.100 | 0.0044 |
| Fig 3A | PBS | 0.100 | 0.4201 |
|  | TNFα | 0.100 | 0.4408 |
|  | H_2_O_2_ | 0.100 | 0.7878 |
|  | PA | 0.100 | 0.4174 |
| Fig 3B | Cleaved casp 8 | 0.100 | 0.5432 |
|  | pRIP1 | 0.100 | 0.6476 |
|  | pRIP3 | 0.100 | 0.8242 |
|  | pMLKL | 0.100 | 0.5016 |
|  | BDNF | 0.100 | 0.2035 |
| Fig 4A | pERK | 0.100 | 0.0443 |
|  | pJNK | 0.100 | 0.4063 |
|  | Cleaved casp 8 | 0.100 | 0.6223 |
|  | pRIP1 | 0.100 | 0.5699 |
|  | pRIP3 | 0.100 | 0.0259 |
|  | pMLKL | 0.100 | 0.2028 |
| Fig 5A | mtROS | 0.100 | 0.2031 |
| Fig 6A | NLRP3 | 0.100 | 0.2111 |
|  | ASC | 0.100 | 0.2111 |
|  | Cleaved casp 1 | 0.100 | 0.8895 |
|  | Cleaved GSDMD | 0.077 | 0.8198 |
| Fig 6B | Casp 1 activity | 0.100 | 0.7257 |
| Fig 6D | NLRP3 | 0.100 | 0.0443 |
|  | ASC | 0.100 | 0.1303 |
|  | Cleaved casp 1 | 0.100 | 0.6204 |
|  | Cleaved IL-1b | 0.100 | 0.1436 |
|  | Cleaved GSDMD | 0.100 | 0.7649 |
| Fig 6F | IL-1b | 0.100 | 0.5075 |
|  | IL-18 | 0.001 | 0.0001 |
| Fig 7B | Nucleated myofiber | 0.100 | 0.0040 |
|  | Mononuclear cell infiltration | 0.063 | 0.8734 |
| Fig 7C | pRIP1 | 0.100 | 0.2084 |
|  | pRIP3 | 0.100 | 0.1717 |
|  | pMLKL | 0.100 | 0.6385 |
|  | NLRP3 | 0.100 | 0.0885 |
|  | ASC | 0.100 | 0.1692 |
|  | Cleaved GSDMD | 0.100 | 0.3298 |
| Fig 7E | pRIP3 | 0.100 | 0.2364 |
|  | pMLKL | 0.100 | 0.1639 |
|  | NLRP3 | 0.100 | 0.2028 |
|  | ASC | 0.100 | 0.1229 |
|  | Cleaved GSDMD | 0.100 | 0.8477 |
| Fig 7F | Mononuclear cell infiltration | 0.100 | 0.1574 |
| Fig 7G | Creatine kinase activity | 0.100 | 0.4890 |
| Fig 7H | Grip strength | 0.100 | 0.4247 |
