# Supplementary material for: Deficiency of muscle-generated brain-derived neurotrophic factor causes inflammatory myopathy through reactive oxygen species-mediated necroptosis and pyroptosis
Source: Redox Biol. 2024 Nov 8;78:103418. doi: 10.1016/j.redox.2024.103418 (PMC11602578; doi:10.1016/j.redox.2024.103418)
Supplement: Multimedia component 3 [file mmc3.docx]

**SUPPLEMENTAL FIGURE LEGEND**

**Fig S1 MBKO mice display histological features of inflammatory myositis without overexpression of autoimmune antigens.**

1. Representative images of the gastrocnemius of 1-, 3-, and 12-month-old female Fl/Fl and MBKO mice (asterisks: myofiber oncosis; yellow arrows: central-nucleated myotube; green arrows: immune cell infiltration; white arrows: myophagocytosis). The scale bar represents 50 μm.
2. Representative images of the gastrocnemius of 1-, 6-, and 12-month-old male Fl/Fl and MBKO mice (yellow arrows: central-nucleated myotube). The scale bar represents 50 μm.
3. Antigen expression in the gastrocnemius of female Fl/Fl and MBKO mice (6 months old) was determined by real-time PCR (Student’s t-test, n=5,).
4. Representative images of the interscapular of 6- and 12-month-old female Fl/Fl and MBKO mice. The scale bar represents 50 μm.

**Fig S2 BDNF-deficiency does not induce apoptosis in muscle.**

1. C2C12 myotubes were infected with Ad-Ctr or Ad-shBDNF, and the apoptosis signaling was determined by Western Blotting. Quantification of the immunoblot is shown in the right panel (Student’s t-test, n=3).
2. Caspase 3 signaling in the gastro of gastrocnemius of female Fl/Fl and MBKO mice (6 months old) was determined by Western blotting.

**Fig S3 Young onset myositis in female MBKO mice.**

1. Necroptosis and pyroptosis signaling in the gastrocnemius of female Fl/Fl and MBKO mice (1 month old female). Quantitation of the immunoblot is shown in the right panel (*: P<0.05, Student’s t-test, n=3).
2. Necroptosis and pyroptosis signaling in the gastrocnemius of male Fl/Fl and MBKO mice (6 months old male). Quantitation of the immunoblot is shown in the right panel (n=4).

**Fig S4 Chronic exercise exaggerates muscle damage in MBKO mice.**

1. Representative images of the gastrocnemius of sedentary (Sed) Fl/Fl and MBKO mice (7 months old female) and those in the animals that have received chronic running training (Exe) for 4 weeks. Mononuclear immune cell infiltration is indicated by the yellow arrows. The black arrows indicate myofibers undergoing severe necrosis. The magnified view of the selected area is shown in the right panel. The scale bars represent 50 μm.
2. Necroptosis signaling in the gastrocnemius of sedentary (Sed) Fl/Fl and MBKO mice (7 months old female) and those in the animals that have received chronic running training (Exe) for 4 weeks. Quantitation of the immunoblot is shown in the right panel (**: P<0.01, ***: P<0.001, two-way ANOVA, n=3).

**Fig S5 Prednisolone (PRDL) treatment did not alleviate the myoinflammation in MBKO mice.**

1. The number of mononuclear immune cell infiltration and myofiber necrosis in the muscle of female MBKO mice (4 months old) after daily injection of PBS or PRDL (5 mg/kg/day) for 4 weeks (*: P<0.05, Student’s t-test, n=4).
2. Creatine kinase activity in the blood of female MBKO mice (4 months old) after daily injection of PBS or PRDL (5 mg/kg/day) for 4 weeks (Student’s t-test, n=5-6).
3. Necroptosis signaling in the gastro of female MBKO mice (4 months old) after daily injection of PBS or PRDL (5 mg/kg/day) for 4 weeks. Quantitation of the immunoblot is shown in the right panel (**: P<0.01, one-way ANOVA, n=3).
4. Expression of *Fgf21* in the liver of female MBKO mice (4 months old) after daily injection of PBS or PRDL (5 mg/kg/day) for 4 weeks (***: P<0.001, Student’s t-test, n=5, 6).

**Fig S6 7,8-DHF treatment did not provoke necroptosis and pyroptosis in skeletal muscle.** Necroptosis and pyroptosis signaling in the gastro of female Fl/Fl mice (9 months old) after H_2_O or 7,8-DHF treatment for 3 months was determined by Western blotting. Quantification of the immunoblot is shown in the right panel (Student’s t-test, n=3).
