# Supplementary figures and images for: Deficiency of muscle-generated brain-derived neurotrophic factor causes inflammatory myopathy through reactive oxygen species-mediated necroptosis and pyroptosis
Source: Redox Biol. 2024 Nov 8;78:103418. doi: 10.1016/j.redox.2024.103418 (PMC11602578; doi:10.1016/j.redox.2024.103418)

**Figure S1**

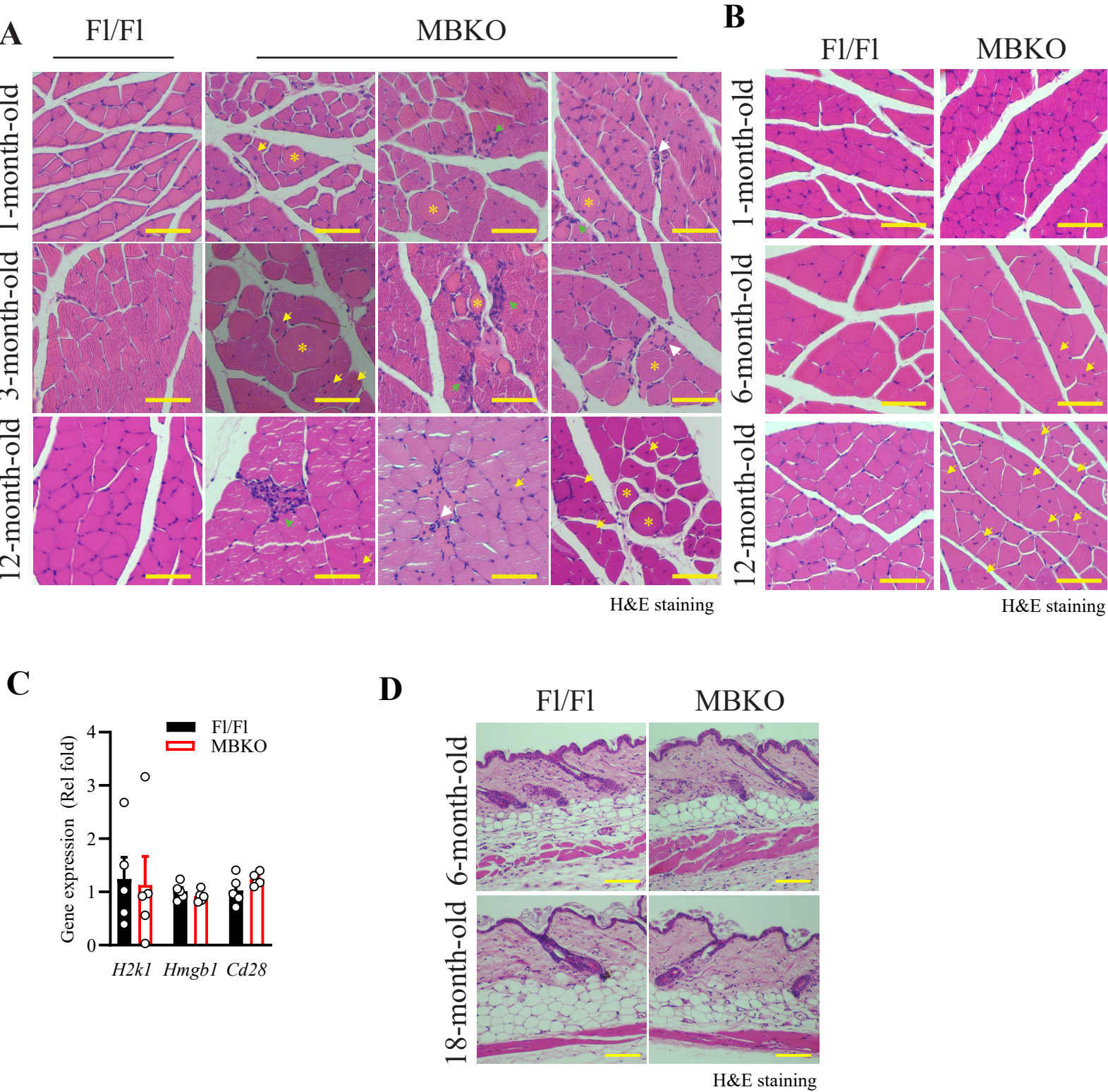

Supplement: Multimedia component 4 [file mmc4.pdf]

**Figure S2**

**A**

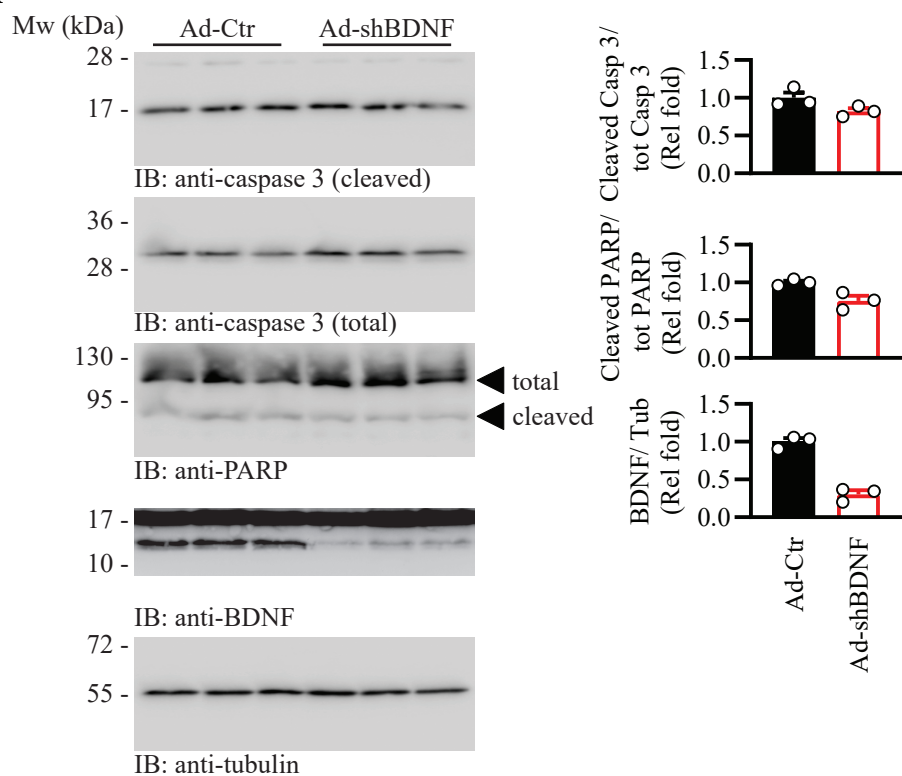

**B**

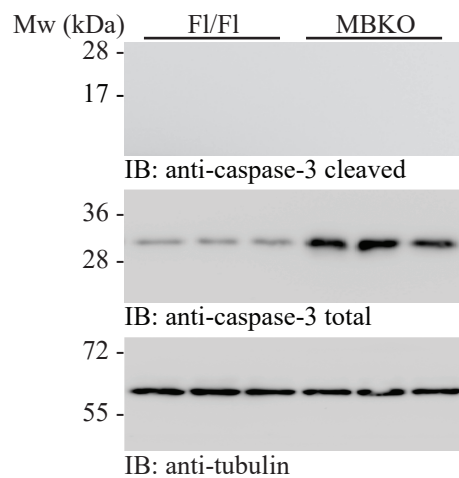

Supplement: Multimedia component 5 [file mmc5.pdf]

# Figure S3

## A

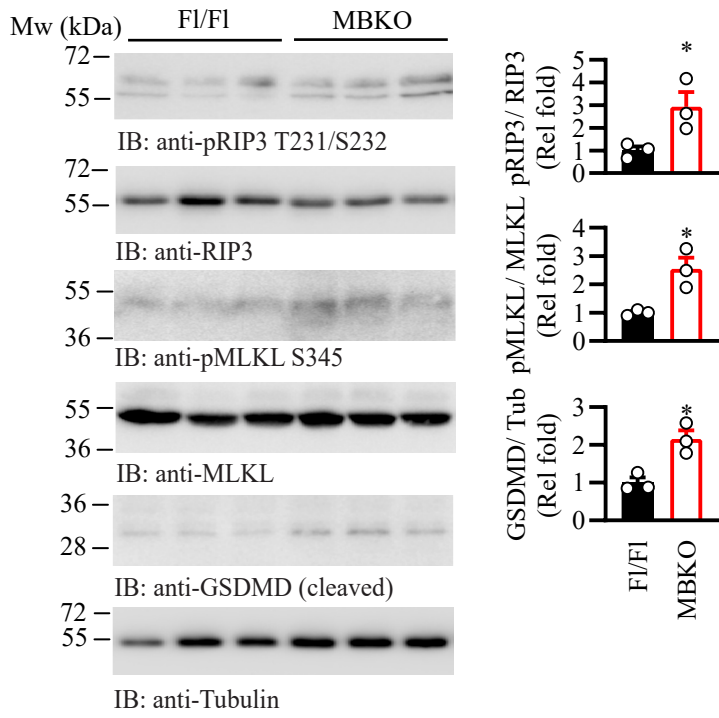

## B

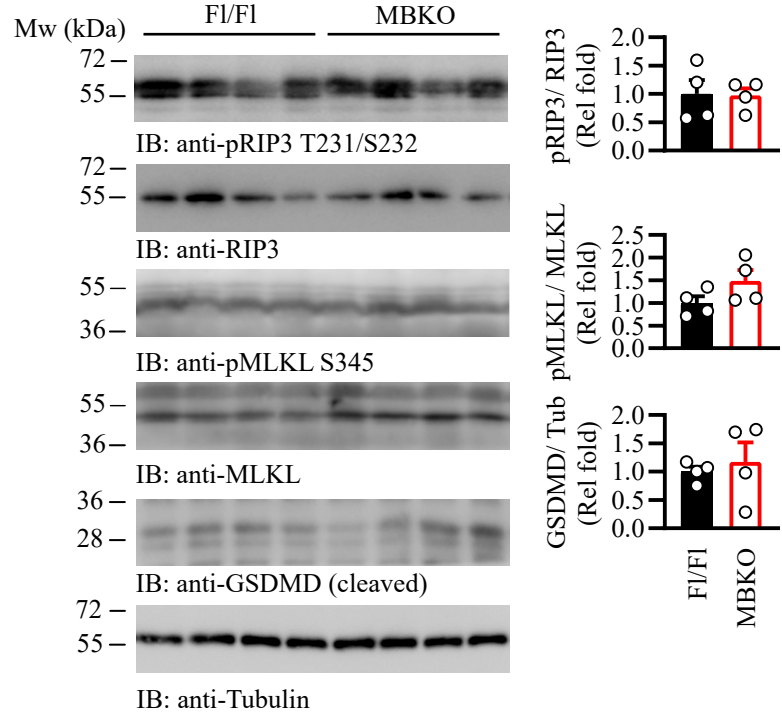

Supplement: Multimedia component 6 [file mmc6.pdf]

**Figure S4**

**A**

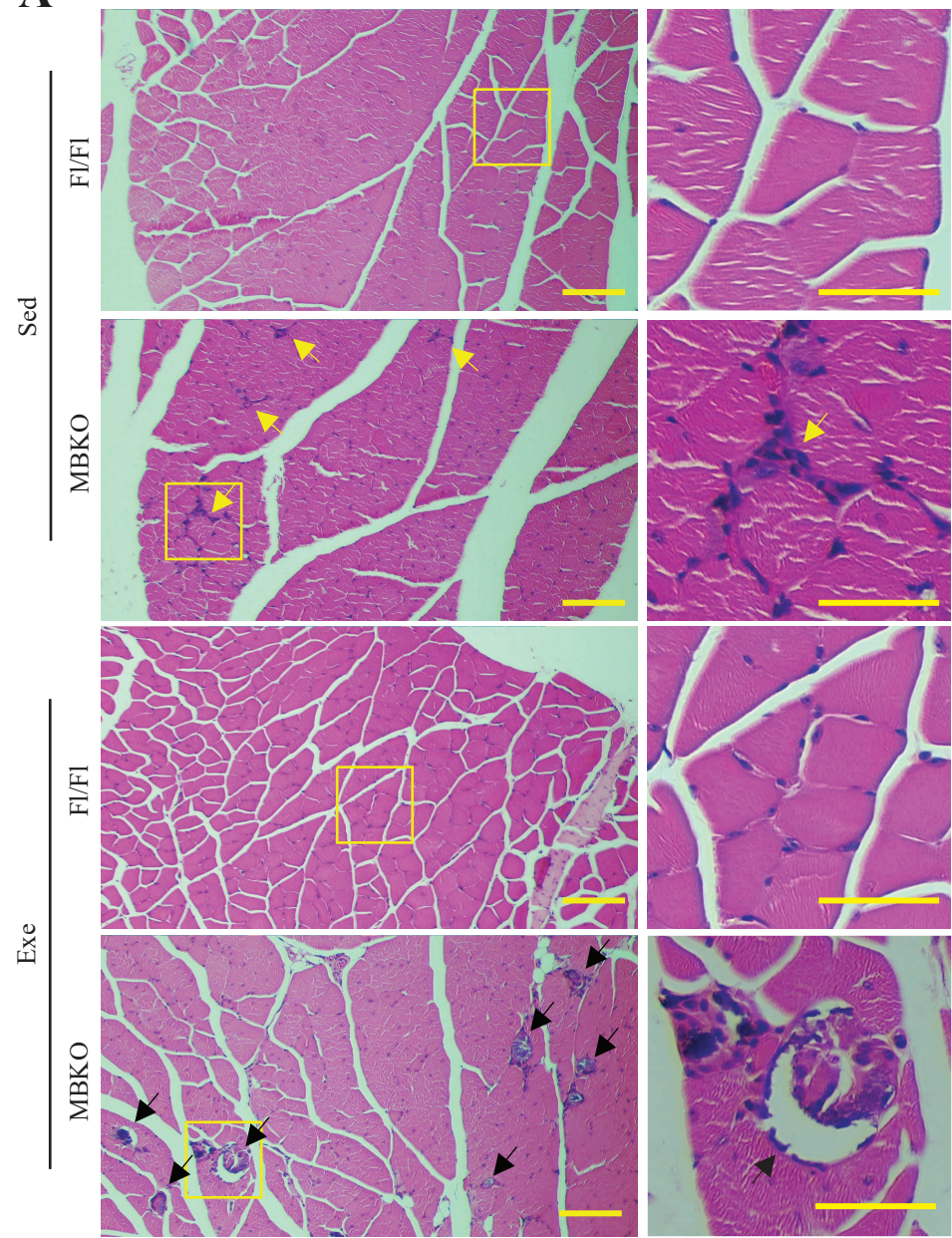

**B**

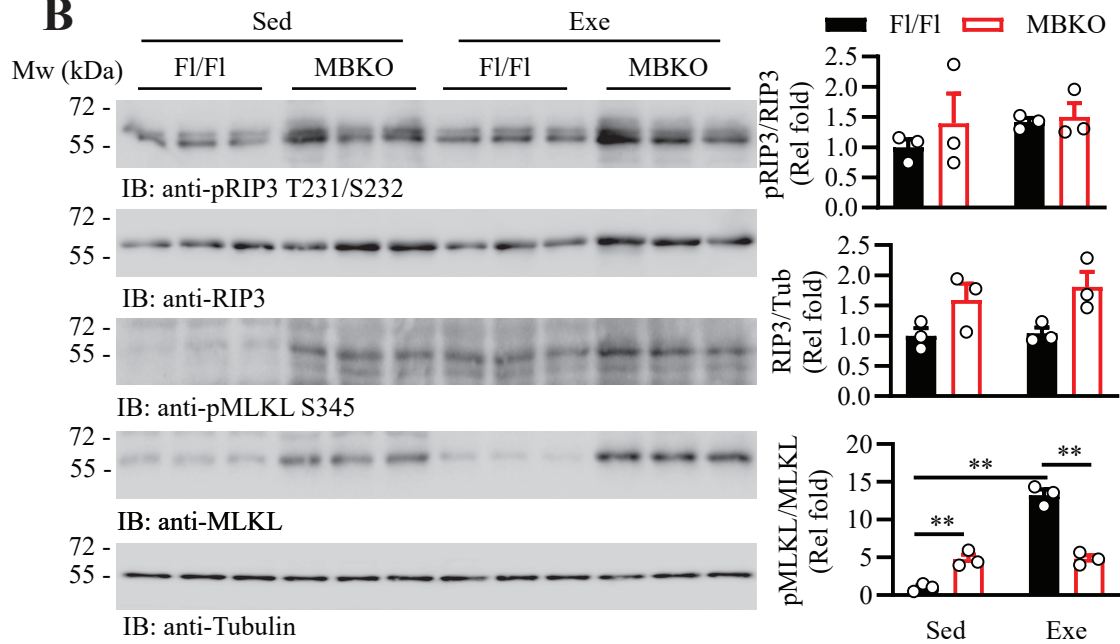

Supplement: Multimedia component 7 [file mmc7.pdf]

Figure S5

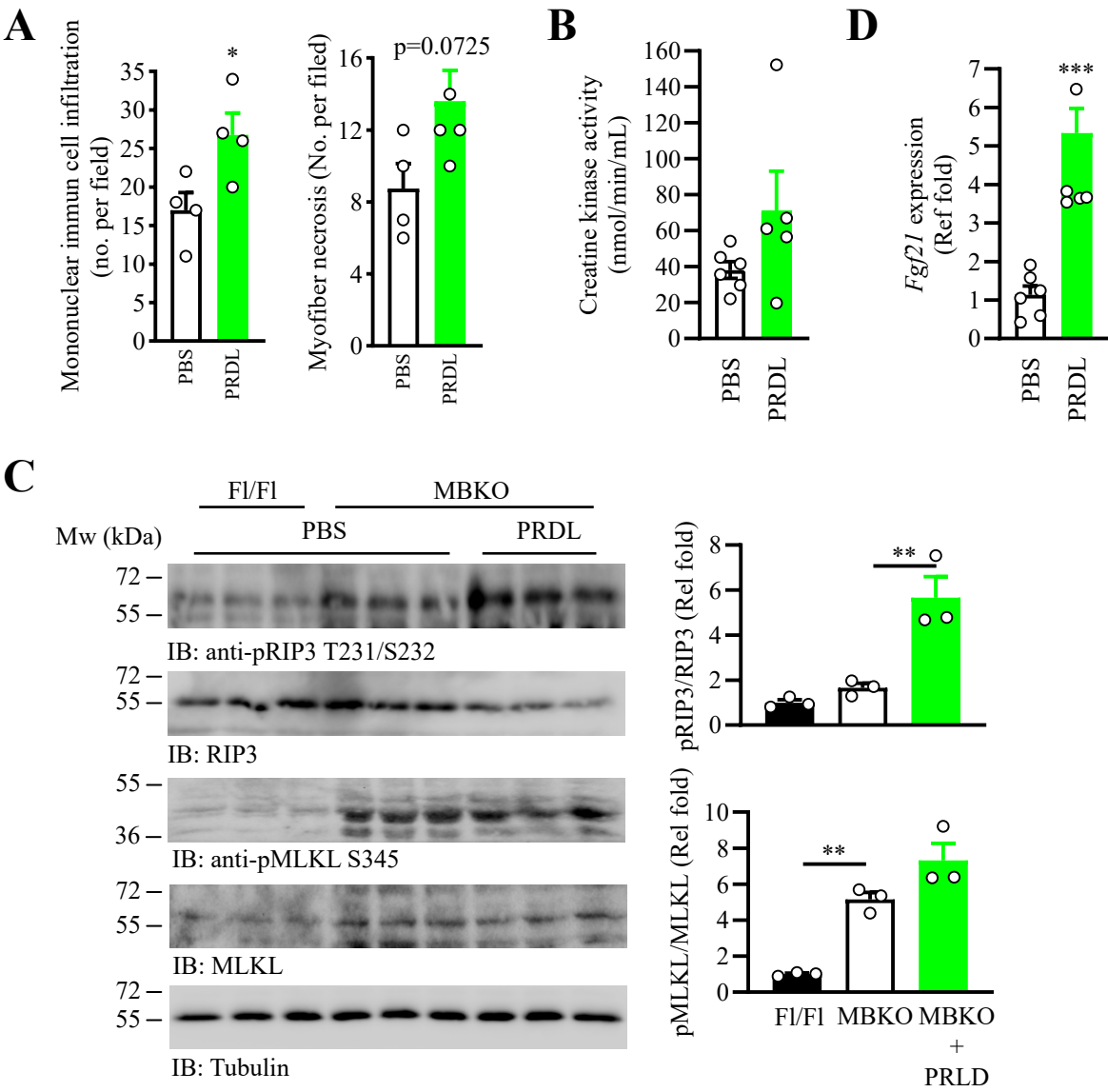

Supplement: Multimedia component 8 [file mmc8.pdf]

Figure S6

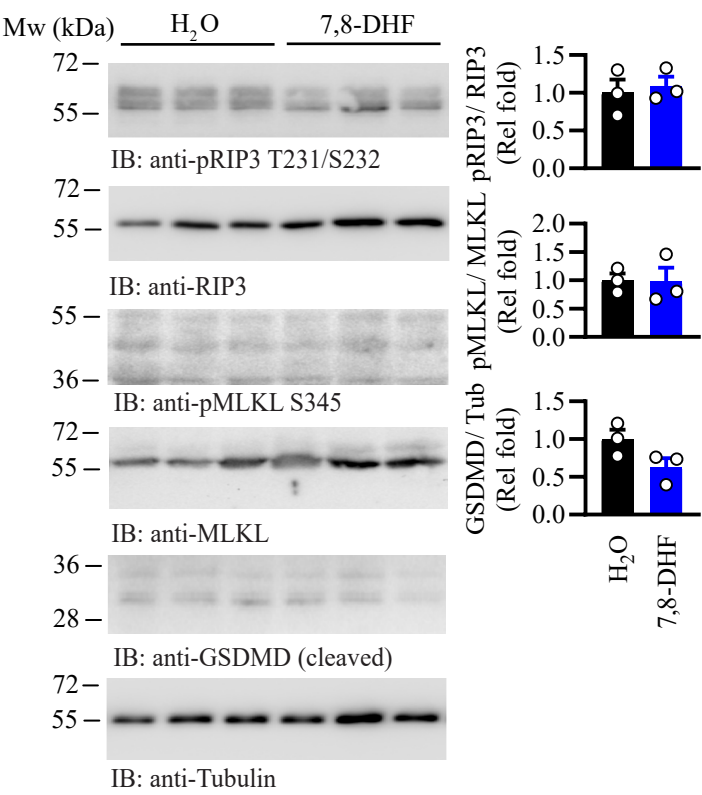

Supplement: Multimedia component 9 [file mmc9.pdf]

Figure S2

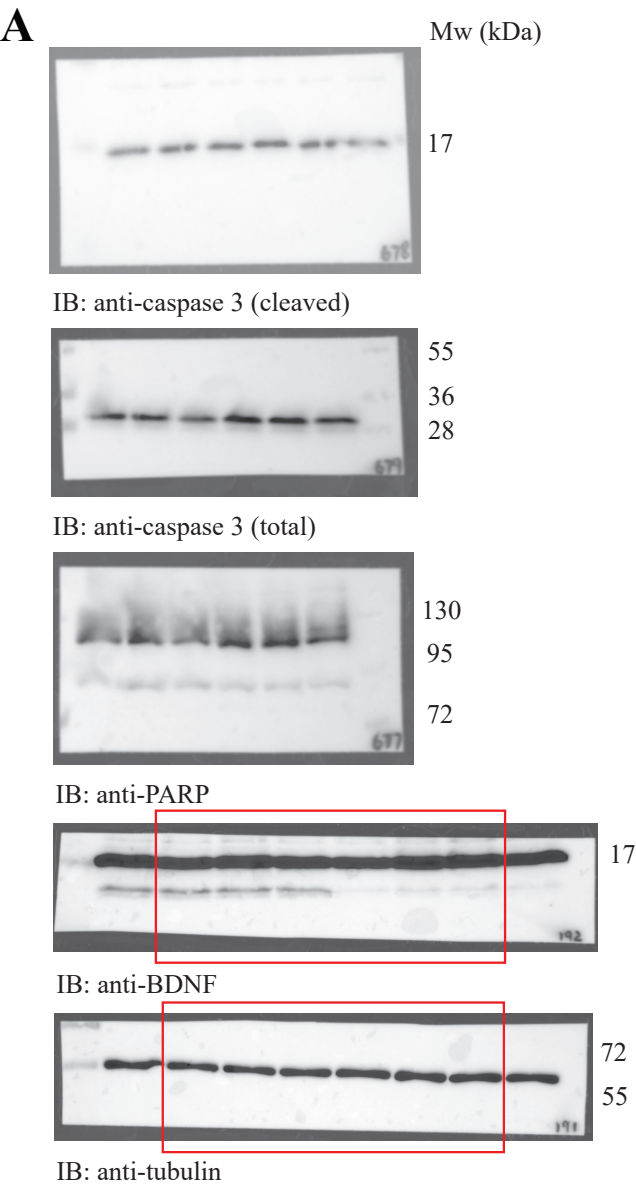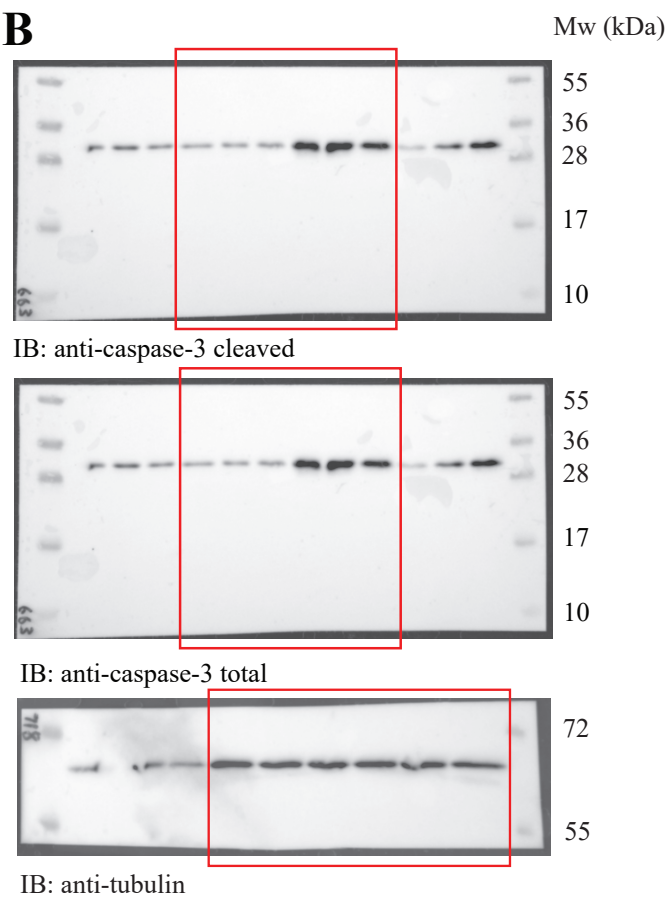

Supplement: Multimedia component 10 [file mmc10.pdf]

Figure 2B

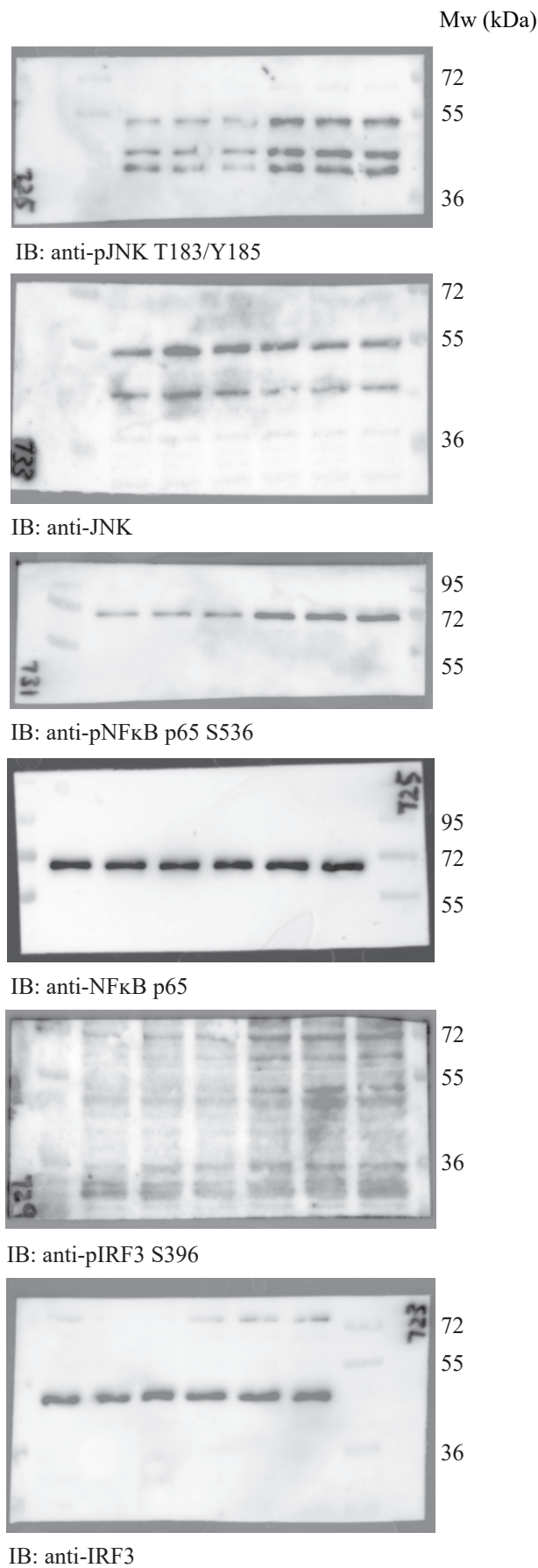

Supplement: Multimedia component 11 [file mmc11.pdf]

Figure S3

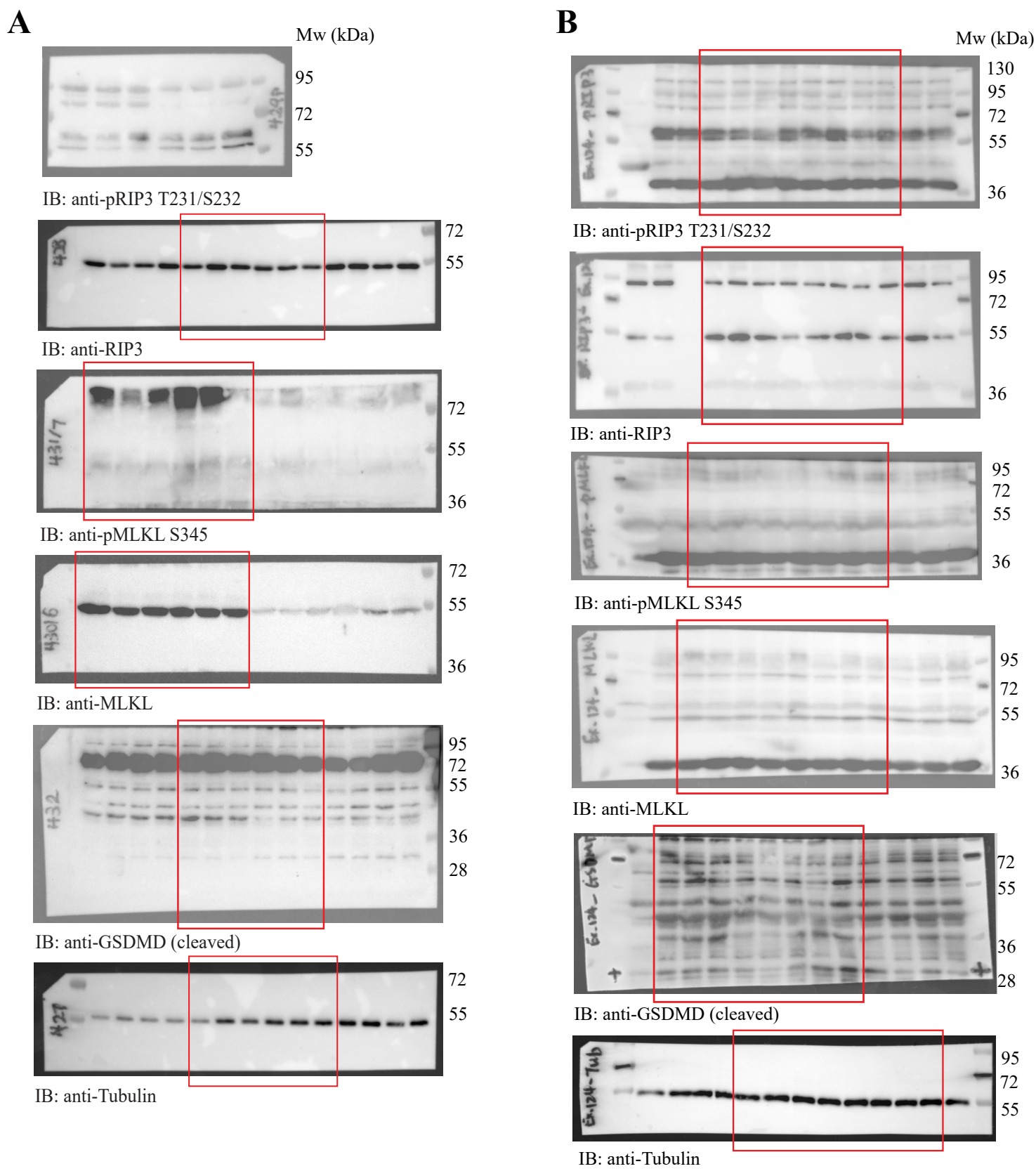

Supplement: Multimedia component 12 [file mmc12.pdf]

Figure 3B

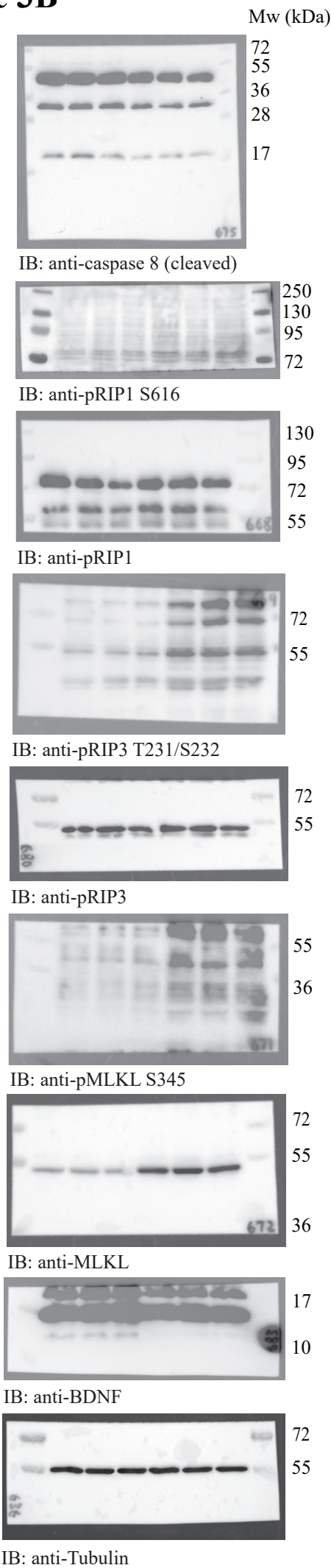

Supplement: Multimedia component 13 [file mmc13.pdf]

Figure 3C

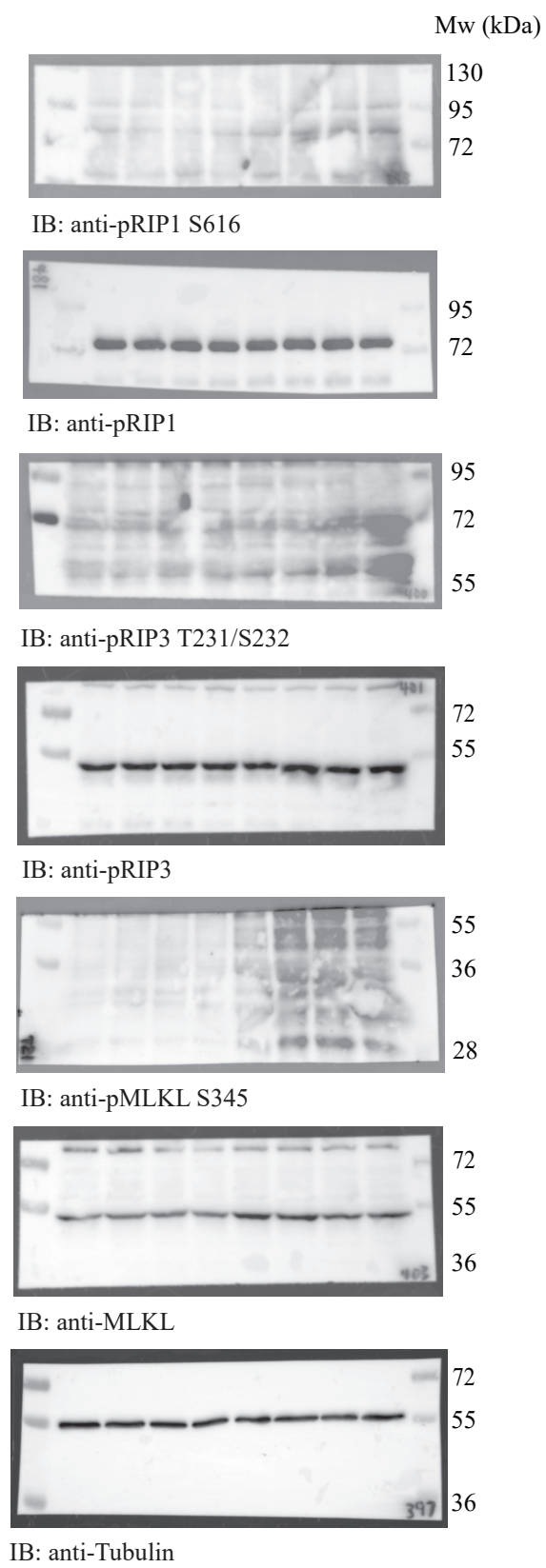

Supplement: Multimedia component 14 [file mmc14.pdf]

Figure S4

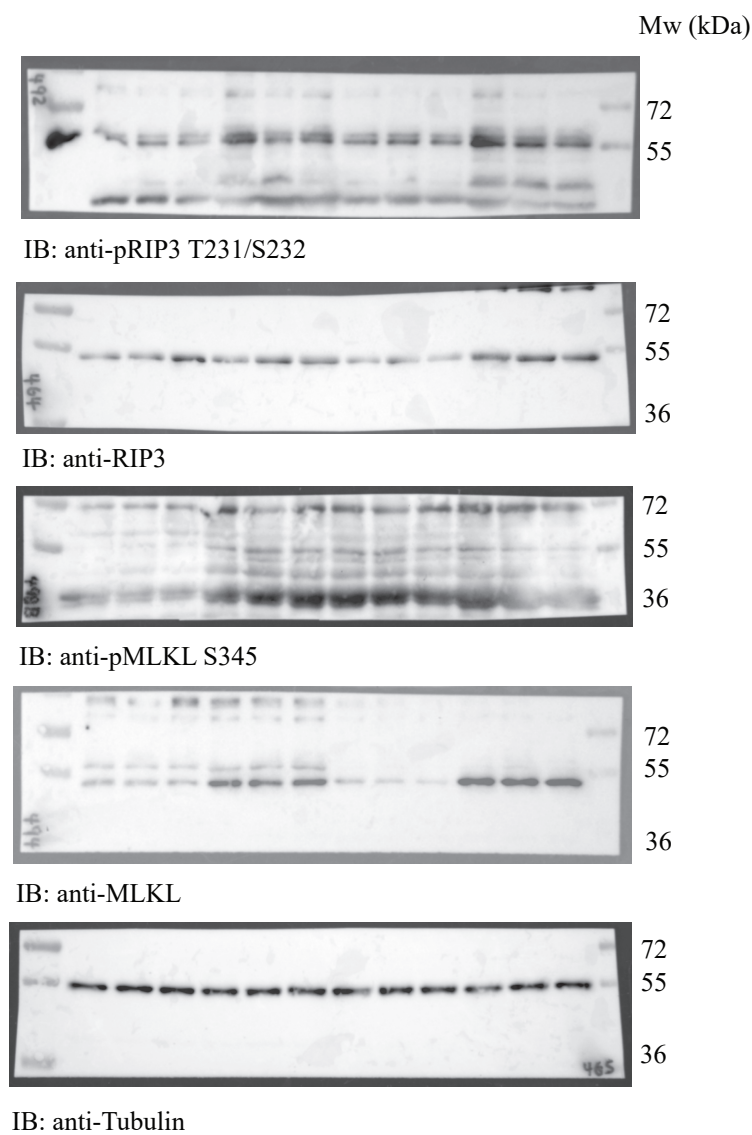

Supplement: Multimedia component 15 [file mmc15.pdf]

Figure 4A

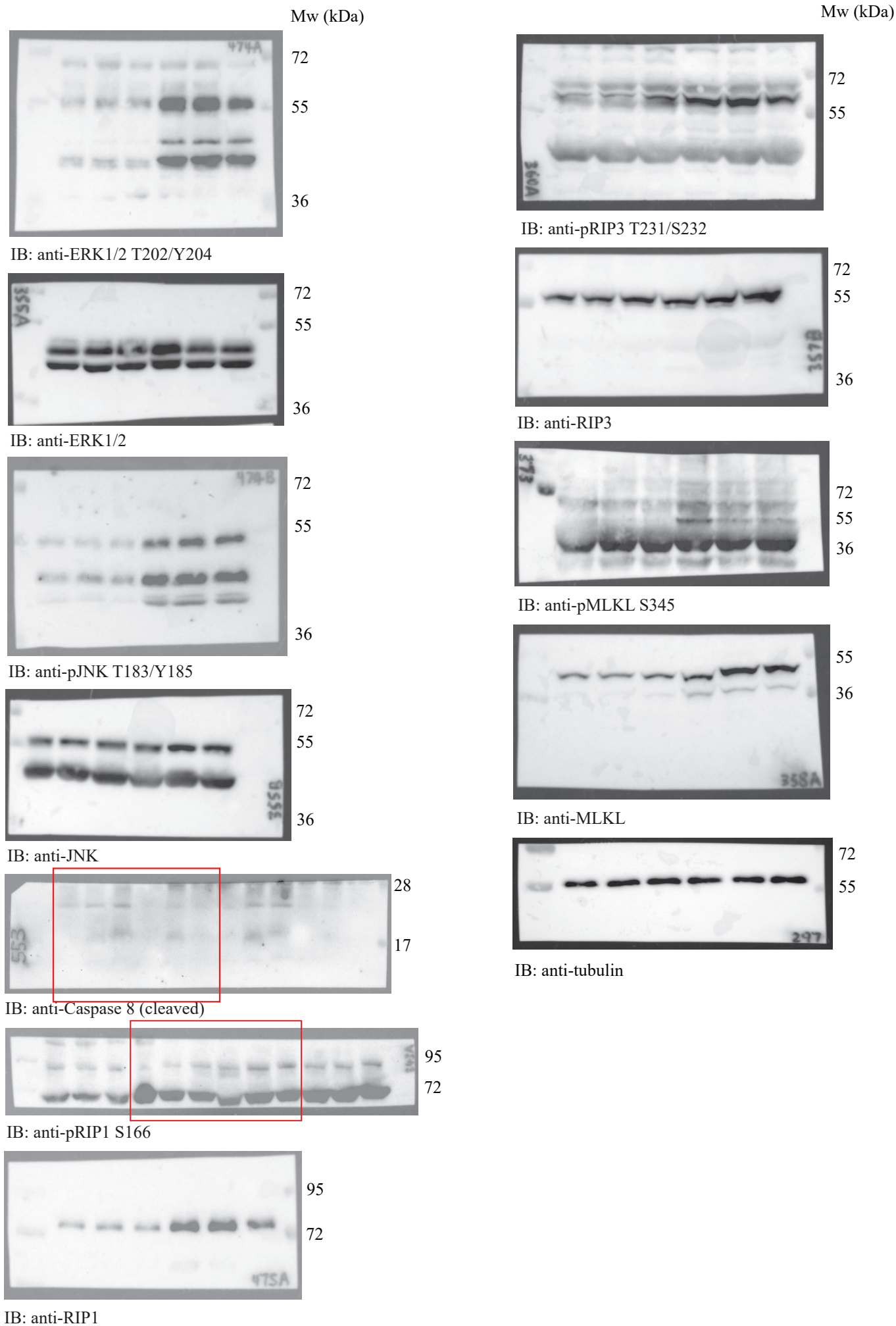

Supplement: Multimedia component 16 [file mmc16.pdf]

Figure 4B

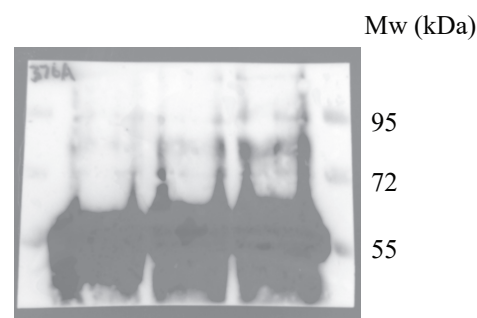

IB: anti-RIP1

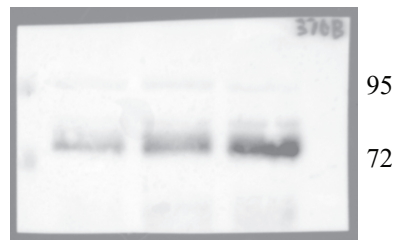

IB: anti-RIP1

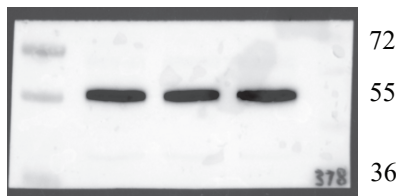

IB: anti-RIP3

Supplement: Multimedia component 17 [file mmc17.pdf]

Figure S5

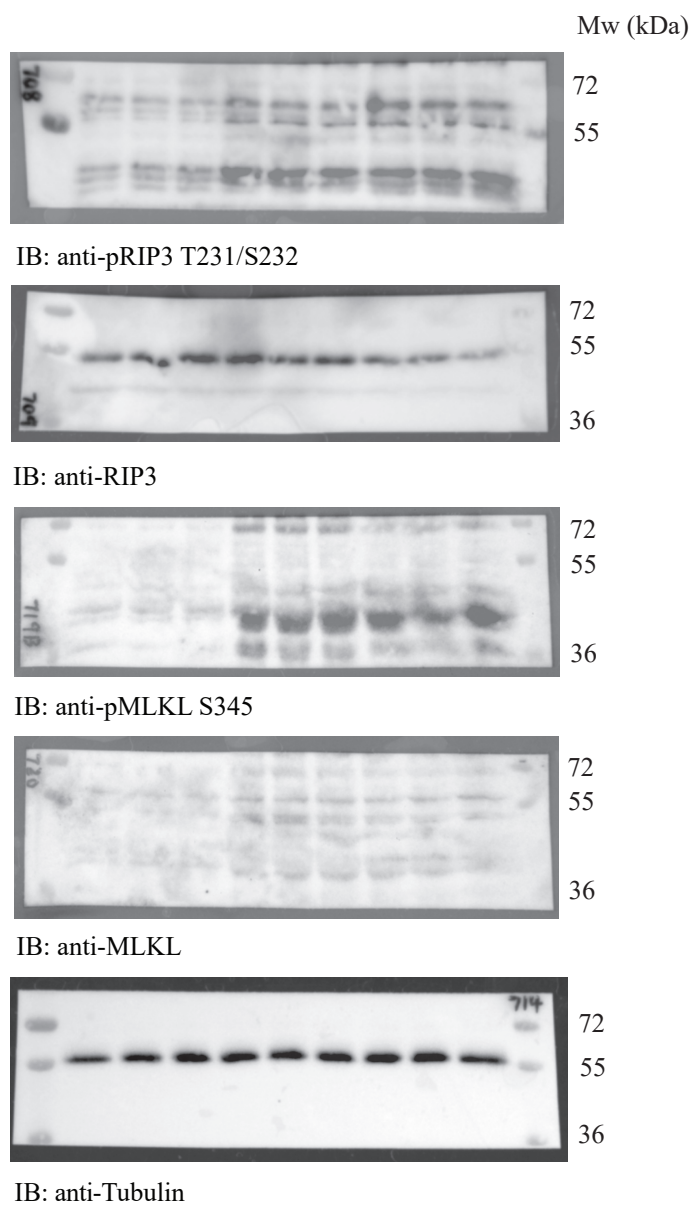

Supplement: Multimedia component 18 [file mmc18.pdf]

Figure 6A

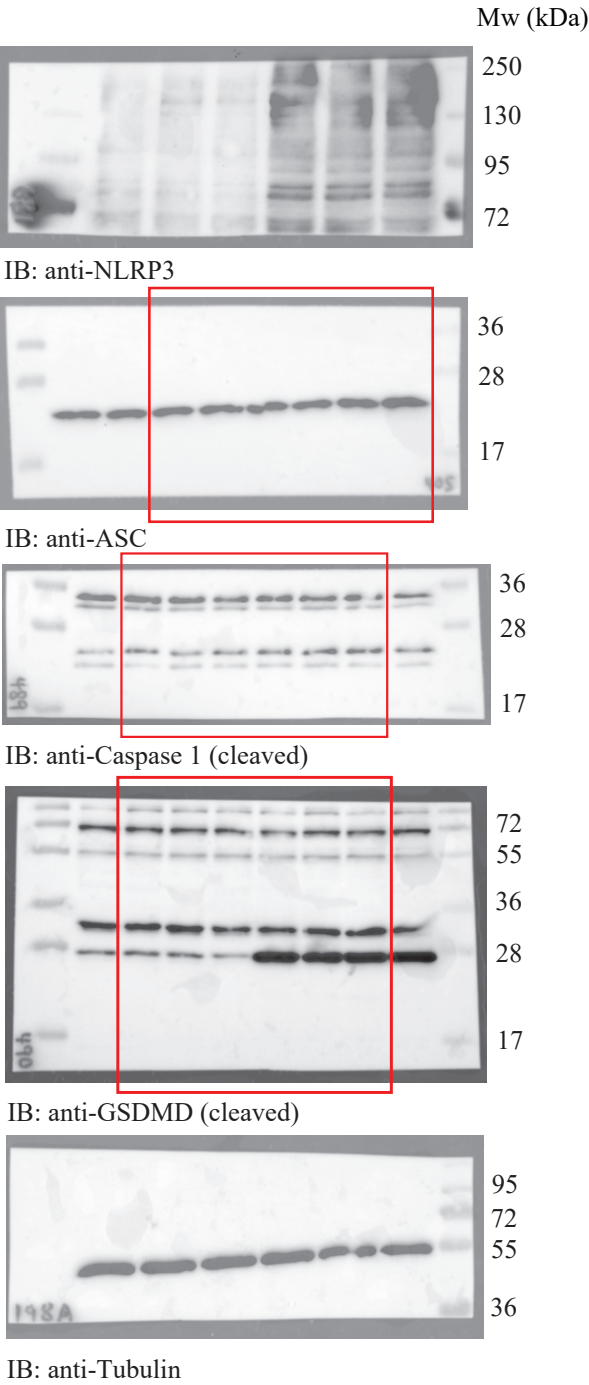

Supplement: Multimedia component 20 [file mmc20.pdf]

Figure 6D

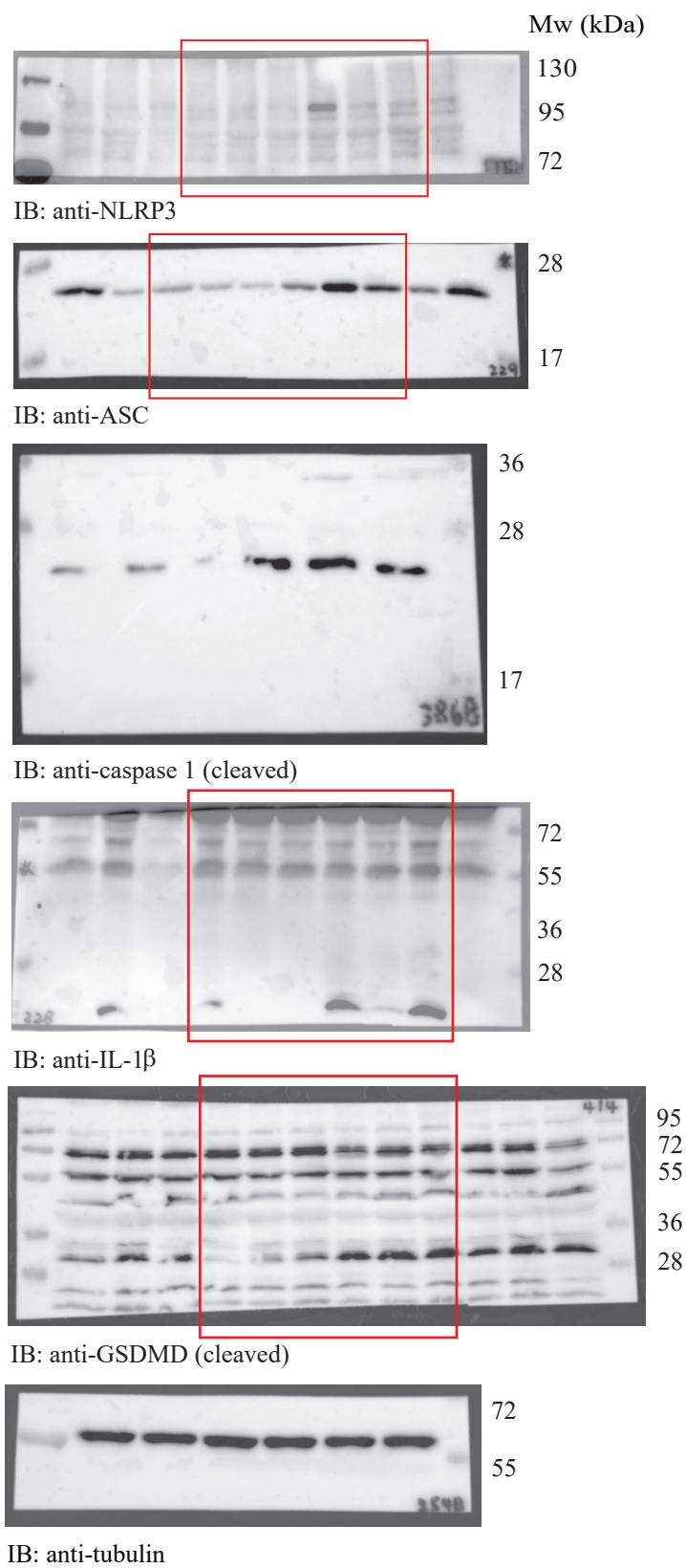

Supplement: Multimedia component 21 [file mmc21.pdf]

### Figure 6G

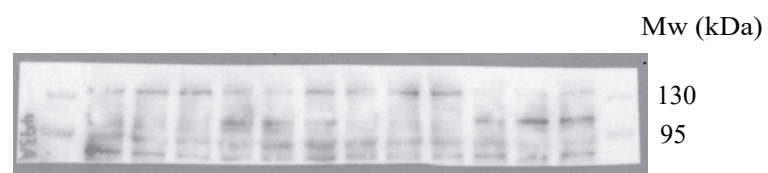

IB: anti-NLRP3

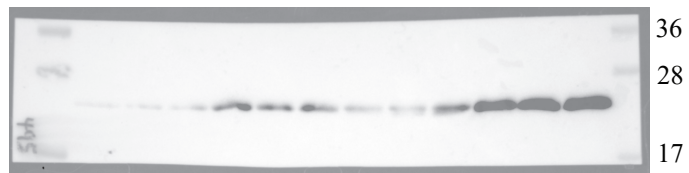

IB: anti-ASC

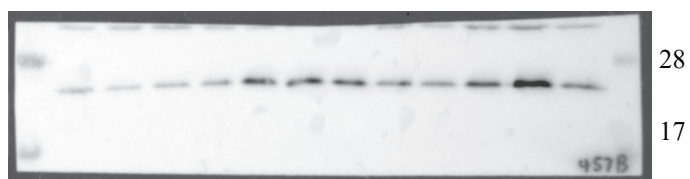

IB: anti-Caspase 1 (cleaved)

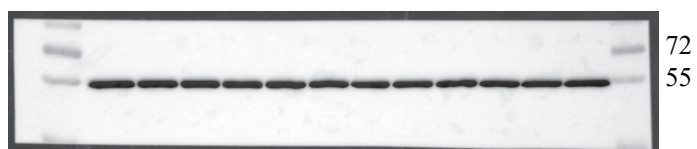

IB: anti-tubulin

Supplement: Multimedia component 22 [file mmc22.pdf]

Figure 7E

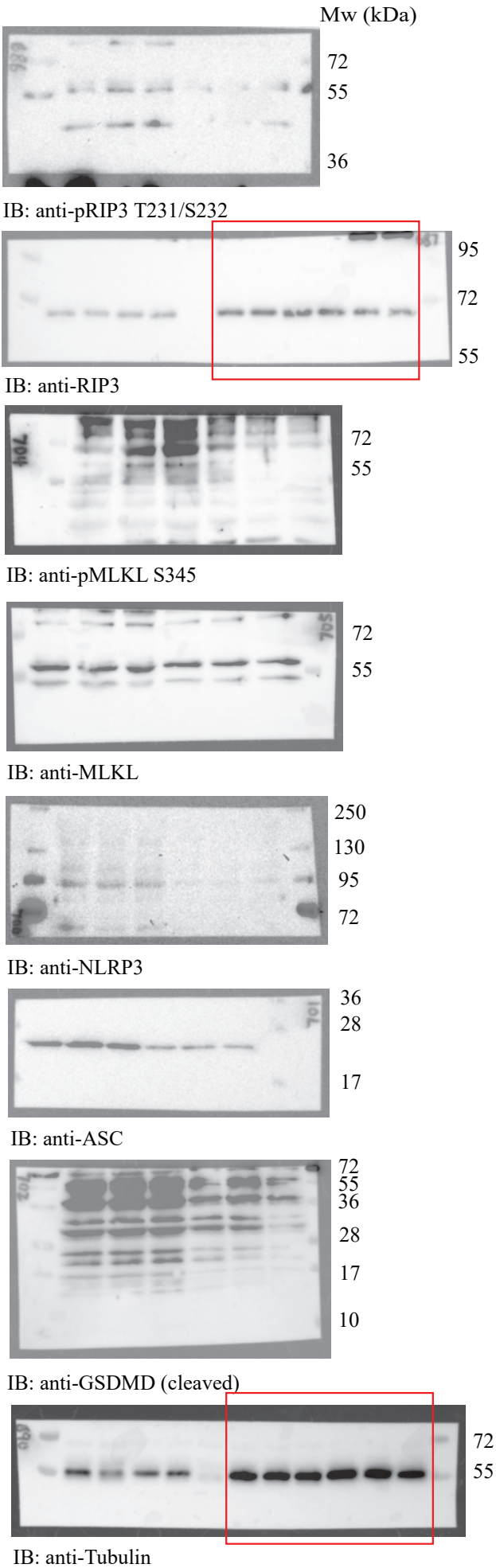

Supplement: Multimedia component 24 [file mmc24.pdf]
