# Supplementary material for: Deficiency of muscle-generated brain-derived neurotrophic factor causes inflammatory myopathy through reactive oxygen species-mediated necroptosis and pyroptosis
Source: Redox Biol. 2024 Nov 8;78:103418. doi: 10.1016/j.redox.2024.103418 (PMC11602578; doi:10.1016/j.redox.2024.103418)
Supplement: Multimedia component 23 [file mmc23.pdf]

Figure 7C

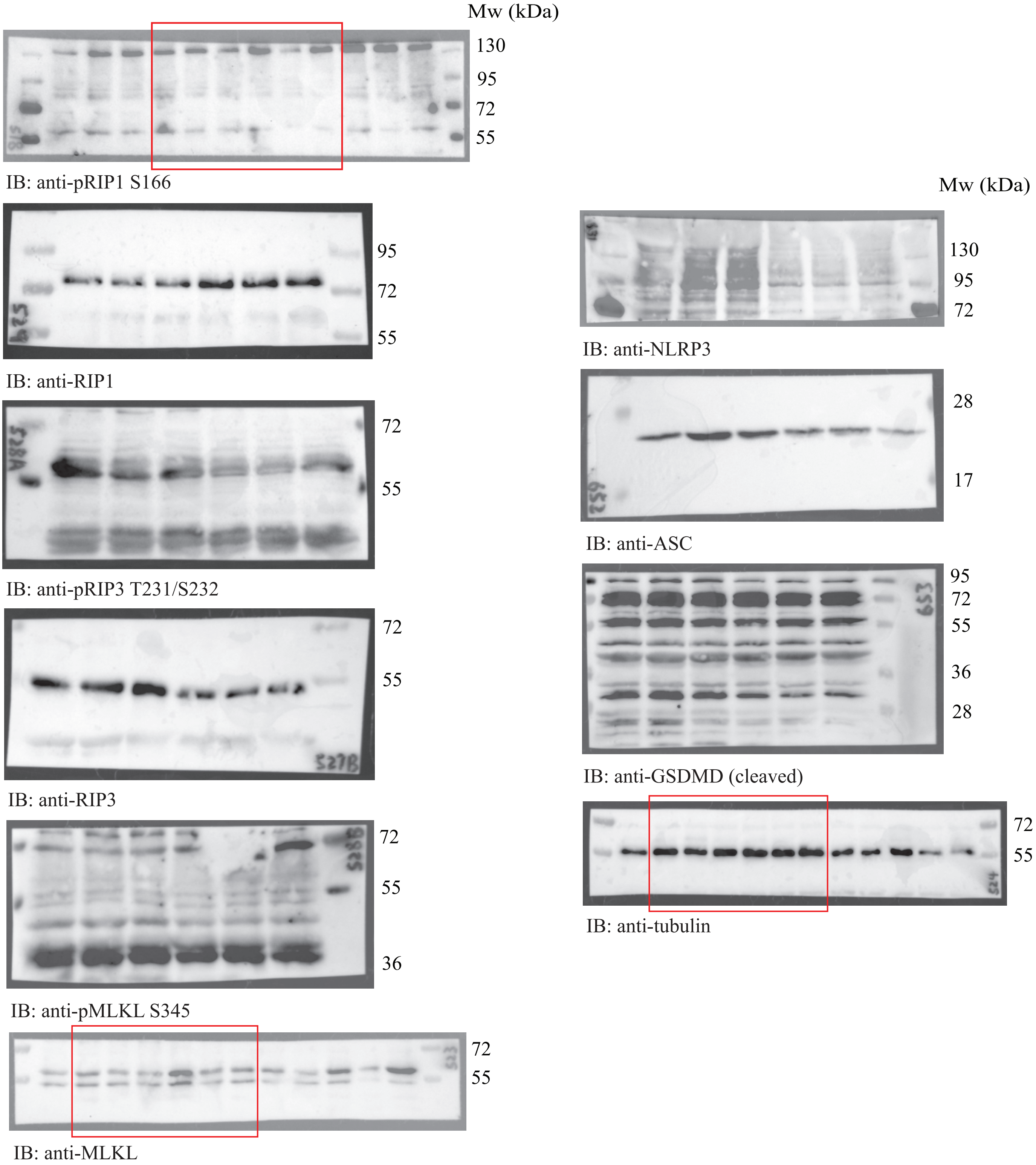
Mw (kDa)
130
95
72
55
IB: anti-pRIP1 S166
95
72
55
IB: anti-RIP1
72
55
IB: anti-pRIP3 T231/S232
72
55
IB: anti-RIP3
72
55
36
IB: anti-pMLKL S345
72
55
IB: anti-MLKL
Mw (kDa)
130
95
72
IB: anti-NLRP3
28
17
IB: anti-ASC
95
72
55
36
28
IB: anti-GSDMD (cleaved)
72
55
IB: anti-tubulin
