# Supplementary material for: Deficiency of muscle-generated brain-derived neurotrophic factor causes inflammatory myopathy through reactive oxygen species-mediated necroptosis and pyroptosis
Source: Redox Biol. 2024 Nov 8;78:103418. doi: 10.1016/j.redox.2024.103418 (PMC11602578; doi:10.1016/j.redox.2024.103418)
Supplement: Multimedia component 25 [file mmc25.pdf]

Figure 7K

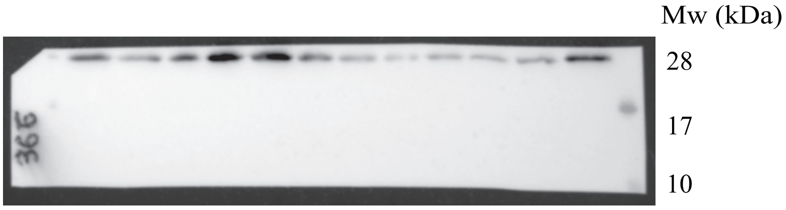

IB: anti-ASC

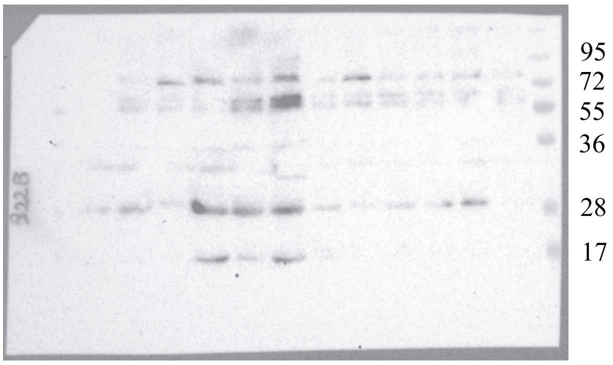

IB: anti-GSDMD (cleaved)

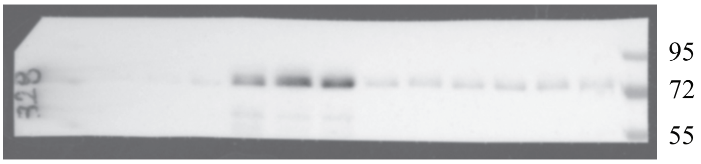

IB: anti-RIP1 S166

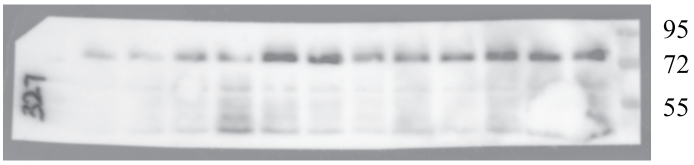

IB: anti-RIP1

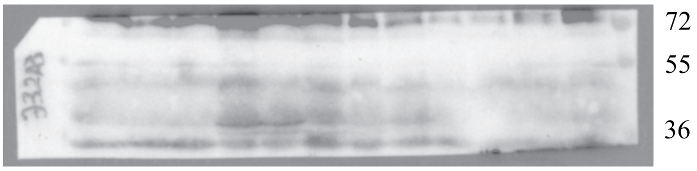

IB: anti-pMLKL S345

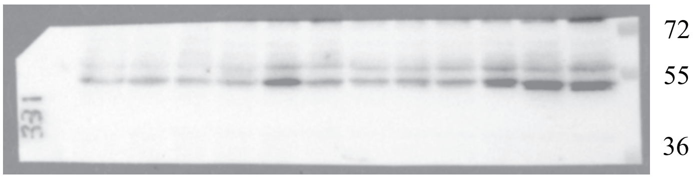

IB: anti-MLKL

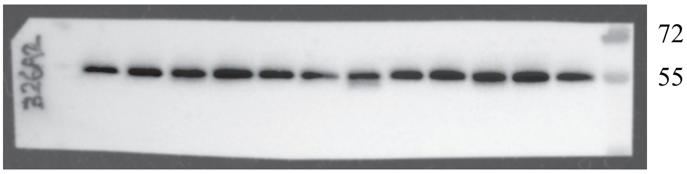

IB: anti-Tubulin
